# Cannabinoid enhancement of lncRNA *MMP25-AS1*/*MMP25* interaction reduces neutrophil infiltration and intestinal epithelial injury in HIV/SIV infection

**DOI:** 10.1172/jci.insight.167903

**Published:** 2023-04-10

**Authors:** Lakmini S. Premadasa, Eunhee Lee, Marina McDew-White, Xavier Alvarez, Sahana Jayakumar, Binhua Ling, Chioma M. Okeoma, Siddappa N. Byrareddy, Smita Kulkarni, Mahesh Mohan

**Affiliations:** 1 Southwest National Primate Research Center, Texas Biomedical Research Institute, San Antonio, Texas, USA.; 2 Department of Pathology, Microbiology and Immunology, New York Medical College, Valhalla, New York, USA.; 3 Department of Pharmacology and Experimental Neuroscience, University of Nebraska Medical Center, Omaha, Nebraska, USA.

**Keywords:** AIDS/HIV, Epithelial transport of ions and water, Molecular pathology, Noncoding RNAs

## Abstract

Intestinal epithelial barrier dysfunction, a hallmark of HIV/SIV infection, persists despite viral suppression by combination antiretroviral therapy (cART). Emerging evidence suggests a critical role for long noncoding RNAs (lncRNAs) in maintaining epithelial homeostasis. We simultaneously profiled lncRNA/mRNA expression exclusively in colonic epithelium (CE) of SIV-infected rhesus macaques (RMs) administered vehicle (VEH) or Δ-9-tetrahydrocannabinol (THC). Relative to controls, fewer lncRNAs were up- or downregulated in CE of THC/SIV compared with VEH/SIV RMs. Importantly, reciprocal expression of the natural antisense lncRNA *MMP25-AS1* (up 2.3-fold) and its associated protein-coding gene *MMP25* (attracts neutrophils by inactivating alpha-1 anti-trypsin/SERPINA1) (down 2.2-fold) was detected in CE of THC/SIV RMs. Computational analysis verified 2 perfectly matched complementary regions and an energetically stable (normalized binding free energy = –0.2626) *MMP25-AS1/MMP25* duplex structure. *MMP25-AS1* overexpression blocked IFN-γ–induced MMP25 mRNA and protein expression in vitro. Elevated MMP25 protein expression in CE of VEH/SIV but not THC/SIV RMs was associated with increased infiltration by myeloperoxidase/CD11b^++^ neutrophils (transendothelial migration) and epithelial CD47 (transepithelial migration) expression. Interestingly, THC administered in combination with cART increased *MMP25-AS1* and reduced MMP25 mRNA/protein expression in jejunal epithelium of SIV-infected RMs. Our findings demonstrate that *MMP25-AS1* is a potentially unique epigenetic regulator of *MMP25* and that low-dose THC can reduce neutrophil infiltration and intestinal epithelial injury potentially by downregulating *MMP25* expression through modulation of *MMP25-AS1*.

## Graphical abstract

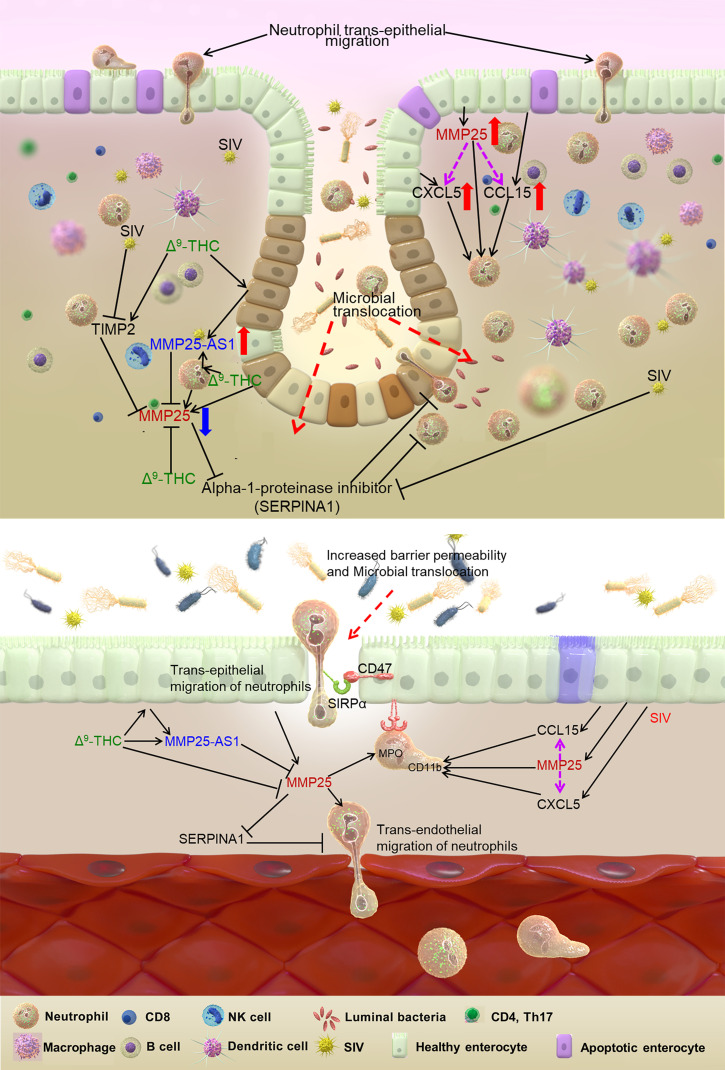

## Introduction

The gastrointestinal (GI) tract is a primary site for HIV/SIV replication, dissemination, and persistence (1). The GI epithelium is a single layer of cells that constitute a semipermeable barrier that allows entry of nutrients and immune sensing, while restricting the movement of potentially pathogenic microbes and microbial antigens. Massive HIV/SIV-induced gastrointestinal CD4^+^ T cell depletion during acute infection causes significant structural and functional damage, thereby disrupting intestinal immune homeostasis. The ensuing dysbiosis and microbial translocation across an impaired intestinal epithelial barrier contribute to local and systemic immune activation/chronic inflammation, a major driver of HIV-associated comorbidities (2, 3). Unfortunately, even early initiation of combination antiretroviral therapy (cART) does not reverse or improve markers of intestinal epithelial damage and microbial translocation (4). Moreover, no FDA-approved drugs that can target the intestinal epithelial barrier are currently available (5). Therefore, a deeper understanding of the mechanisms driving GI epithelial barrier breakdown is needed for the development of effective therapeutic strategies to attenuate chronic intestinal inflammation and systemic immune activation in people living with HIV (PLWH).

Although the molecular mechanisms underlying GI epithelial dysfunction remain ill-defined, emerging evidence has demonstrated that epigenetic mechanisms [aberrant DNA methylation, histone modification, and noncoding RNAs, microRNAs (miRNAs), and long noncoding RNAs (lncRNAs)] significantly contribute to epithelial dysfunction in inflammatory bowel disease (IBD) and irritable bowel syndrome (6–8). Recently, lncRNAs have emerged as a new class of epigenetic regulators of intestinal epithelial homeostasis with the potential to regulate intestinal mucosal response, secretions, and barrier function. LncRNAs have been reported to regulate gene expression at different levels, including chromatin modification, pre-mRNA transcription and splicing, and protein translation. In this context, emerging evidence shows the immense potential of lncRNAs to regulate innate immunity, viral replication, and development and progression of inflammatory diseases by modulating the expression of multiple genes and activation of signaling pathways (9–11). LncRNAs normally function by enhancing or inhibiting the expression of neighboring protein-coding genes (12). Nevertheless, the role of lncRNAs in HIV/SIV-induced intestinal epithelial dysfunction and, more importantly, the possibility of modulating their activity to reverse intestinal epithelial dysfunction remain unknown and unaddressed.

New findings from a recent clinical trial show that oral phytocannabinoids are safe and well tolerated by PLWH (13). Importantly, oral cannabinoids do not alter hematology/biochemistry profiles, CD4 count, or CD4/CD8 ratio, and HIV suppression remains stable (13), suggesting a potentially safe strategy to reduce chronic immune activation in PLWH. Moreover, dronabinol, an FDA-approved synthetic Δ-9-tetrahydrocannabinol (THC) drug, is prescribed for the symptomatic treatment of chemotherapy-associated nausea and vomiting in patients with cancer and treatment of anorexia (to stimulate appetite) in PLWH. Two recent studies (14) summarizing results from numerous randomized controlled clinical trials that utilized synthetic THC (dronabinol, nabilone) and natural phytocannabinoid combinations (nabiximols and cannabidiol) in providing symptomatic relief in a variety of chronic conditions concluded that cannabinoids are effective therapeutics for several medical indications and that future studies should be based on their specific pharmacological properties. Despite these encouraging results, it is equally important for future preclinical research to use biologically relevant animal (nonhuman primate) models to determine molecular mechanisms associated with phytocannabinoid action to improve therapeutic applications. Our published studies demonstrated the ability of long-term low-dose THC to reduce HIV/SIV infection–induced T cell activation/proliferation, to increase the frequency of CD163^+^ macrophages and enhance tight junction protein expression in the intestine (15, 16), and to modulate immune/inflammatory responses in the oral cavity and the salivary microbiome (17, 18) even in the absence of cART. Given these findings and the lack of availability of intestinal epithelial barrier targeting agents, there is a strong need to further investigate the effects and mechanisms of action of long-term THC on the intestinal epithelial compartment at doses that produce beneficial but no detectable adverse effects.

Based on the emerging evidence of the role of lncRNAs in intestinal diseases (19), we hypothesized that HIV/SIV-induced GI epithelial dysfunction is associated with lncRNA dysregulation. Given the high expression of cannabinoid receptor 1 (CB1R) and 2 (CB2R) in the intestinal epithelium (20), we further hypothesized that THC can successfully modulate HIV/SIV-induced inflammation-driven lncRNA dysregulation in the latter compartment. Moreover, such studies are challenging to perform in humans because of the very limited or lack of availability of postmortem whole intestinal tissues for molecular studies. The limited number of colonic pinch biopsies (3 to 5 pinches) that can be collected from human patients does not yield a sufficient number of colonic epithelial cells to perform molecular studies. The availability of the biologically relevant SIV-infected rhesus macaque (RM) model of HIV infection offsets these limitations as it enables the collection of intact intestinal segments antemortem (21) and at necropsy for isolation of sufficient quantities of pure epithelial cells for in-depth molecular studies. Using a potentially unique and robust lncRNA/mRNA microarray platform, we report a lncRNA-mediated mechanism associated with intestinal epithelial barrier injury and neutrophil infiltration and its modulation by phytocannabinoids in HIV/SIV infection.

## Results

### HIV/SIV infection is characterized by significant dysregulation of lncRNA expression in the colonic epithelium.

**Figure 1:**
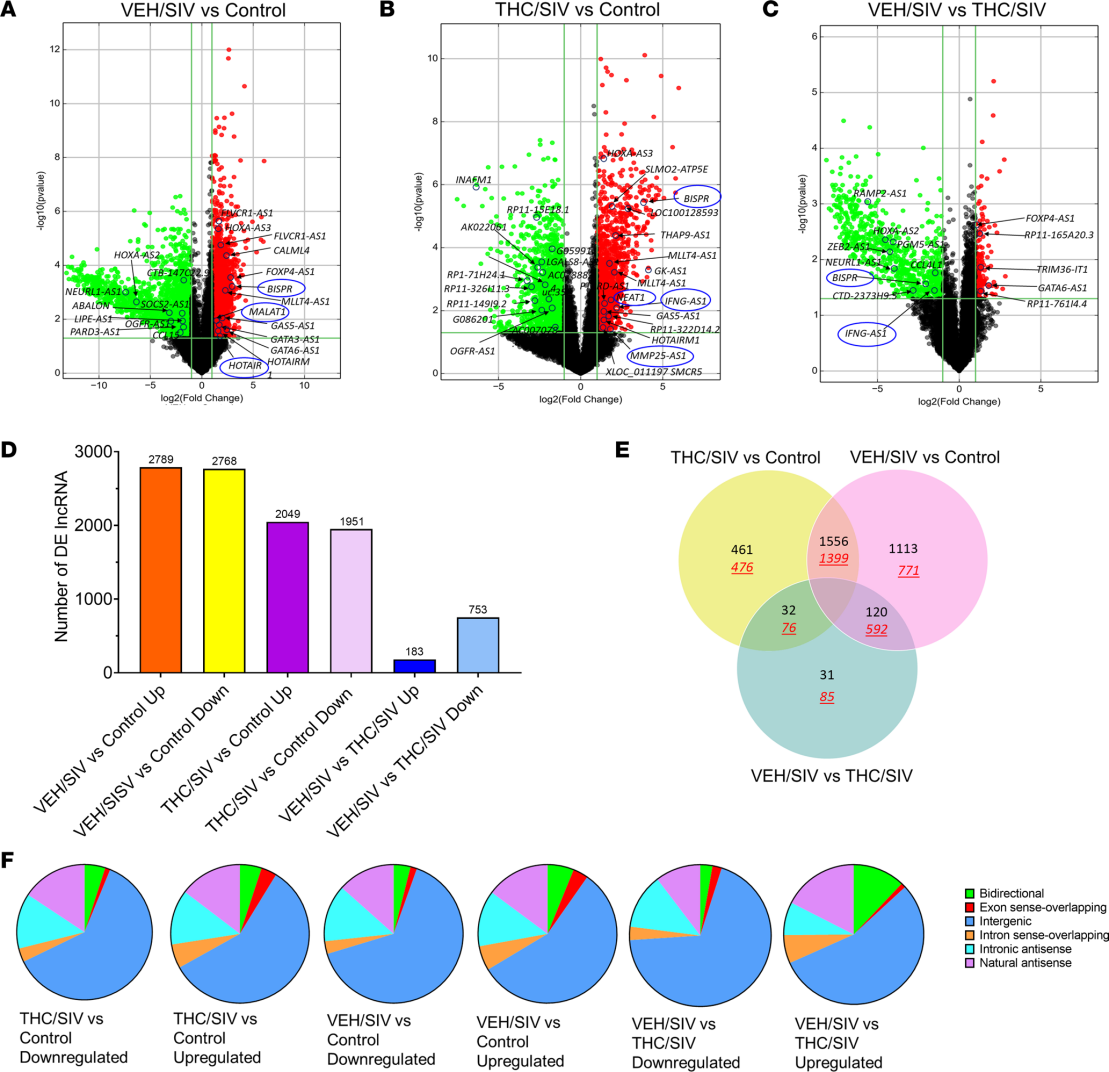
Changes in the colonic epithelial lncRNA landscape in response to HIV/SIV infection and THC administration. Volcano plots show the relationship between fold-change (*x* axis) and statistical significance (*y* axis) of DE lncRNAs in the CE of VEH/SIV (*n* = 5) (**A**) and THC/SIV (*n* = 6) (**B**) relative to control (*n* = 6) RMs and VEH/SIV relative to THC/SIV RMs (**C**). The negative log of statistical significance (*P* value) (base 10) is plotted on the *y* axis, and the log of the fold-change (base 2) is plotted on the *x* axis. Notable DE lncRNAs are shown in the volcano plots. The total number of DE (up- and downregulated) lncRNAs in each treatment group is shown (**D**). Venn diagram illustrates the overlap between the DE lncRNAs (black, upregulated; red, downregulated) in the 3 treatment groups (**E**). Distribution of the 6 classes of lncRNAs based on the genomic region of origin within each comparison (**F**).

Viral inoculum, plasma and colon viral loads, and colon histopathology information for all vehicle/SIV (VEH/SIV) and THC/SIV RMs is provided in Table 1 (15). To determine the role of lncRNAs in HIV/SIV-induced intestinal epithelial dysfunction, we profiled and identified differentially expressed (DE) lncRNAs based on a log_2_ fold-change (FC) cutoff of >2.0 for upregulated lncRNAs and FC < –2.0 for downregulated lncRNAs with *P* < 0.05. The log_2_ FC values and –log_10_ *P* values are plotted and presented as volcano plots for DE lncRNAs in colonic epithelium (CE) of VEH/SIV (Figure 1A) and THC/SIV (Figure 1B) relative to uninfected control RMs and DE lncRNAs in VEH/SIV (Figure 1C) relative to THC/SIV RMs. As evident in Figure 1, A and D, relative to uninfected controls, a greater number (1.4-fold higher) of differentially upregulated lncRNAs were identified in CE of VEH/SIV (*n* = 2,789) compared with THC/SIV (*n* = 2,049) RMs. Similarly, the number of significantly downregulated lncRNAs also showed an identical trend with a greater number (~1.4-fold higher) of lncRNAs showing significantly reduced expression in CE of VEH/SIV (*n* = 2,768) compared with THC/SIV (*n* = 1,951) RMs. At 6 months postinfection (MPI), when comparing VEH/SIV and THC/SIV, 183 and 753 lncRNAs showed significantly increased and decreased expression, respectively. Overall, THC administration decreased the number of DE up- and downregulated lncRNAs in CE of chronically SIV-infected RMs. Important lncRNAs up- and downregulated in VEH/SIV versus controls (Supplemental Tables 1 and 2; supplemental material available online with this article; https://doi.org/10.1172/jci.insight.167903DS1; respectively), THC/SIV versus controls (Supplemental Tables 3 and 4, respectively), and VEH/SIV versus THC/SIV (Supplemental Tables 5 and 6, respectively) RMs are shown in the online supplement.

Although the expression of several DE lncRNAs overlapped among the 3 treatment groups, a Venn diagram analysis (Figure 1E) verified that the VEH/SIV group displayed a substantially higher number of total unique DE lncRNAs compared with the THC/SIV group. In addition, the Venn diagram also shows the number of DE lncRNAs that either were unique to each group or overlapped among the 3 groups (Figure 1E).

Based on origin, all DE lncRNAs identified in the present study belonged to the following 6 established functional types: intergenic, bidirectional, intronic antisense, exon sense-overlapping, intron sense-overlapping, and natural antisense. The pie charts in Figure 1F show the distribution of the 6 classes of lncRNAs within each comparison. Interestingly, most of the DE lncRNAs in all 3 comparisons belonged to the intergenic group (55%–69%) followed by natural antisense (10%–17%) and intronic antisense (7%–13%), both of which were also well represented. In contrast, bidirectional (2%–12%), intron sense overlapping (2%–6.5%) and exon sense overlapping (1%–3.6%) lncRNAs represented a small percentage. In both THC/SIV and VEH/SIV groups, the distribution of the different lncRNA types showed more or less the same pattern.

### THC reciprocally regulates the expression of lncRNAs and their corresponding protein-coding genes associated with inflammation and intestinal epithelial barrier function.

Relative to uninfected controls, fewer DE lncRNAs were detected in CE of THC/SIV compared with VEH/SIV RMs. Importantly, the well-characterized *HOTAIR*, and the expression of several lncRNAs previously associated with inflammation, namely *MALAT-1*, *GATA6-AS1*, *GATA3-AS1*, and *SPRY-IT1* (22–24), were exclusively upregulated in CE of VEH/SIV RMs. Similarly, lncRNAs previously associated with epithelial immune response, namely *NEAT1*, *IFNG-AS1*, and *BISPR* (24–26), were upregulated only in THC/SIV RMs. Interestingly, *IFNG-AS1* was upregulated (FC = 4.7) in CE of THC/SIV RMs compared with uninfected controls, while its expression was significantly downregulated (FC = 2.8) (*P* = 0.04) in VEH/SIV RMs compared with THC/SIV RMs.

Notably, the downregulation of 26 natural antisense lncRNAs was associated with the upregulation of their corresponding protein-coding genes in CE of THC/SIV RMs (Supplemental Table 7). Most notably, a natural antisense lncRNA, *MAGI2-AS3*, located in proximity to the epithelial barrier maintenance gene *MAGI2*, was significantly downregulated (FC = 3.2) in the CE of THC/SIV RMs. Conversely, *MAGI2* mRNA was significantly upregulated (FC = 2.7) in CE of THC/SIV RMs. Other important genes that showed reciprocal upregulation to their corresponding natural antisense lncRNAs included *CCL15* and *ITGA6*. *CCL15*, an important gene reported to be upregulated in the intestinal epithelium of patients with IBD (27) and known to play a critical role in neutrophil infiltration (neutrophil chemotaxis) (28), was significantly upregulated in VEH/SIV RMs (FC = 9.5), while its related natural antisense lncRNA RP11-104J23.1 was downregulated (FC = 3.6). However, *CCL15* was also upregulated in THC/SIV RMs, but with a lower FC of 3.6 compared with VEH/SIV RMs, while its lncRNA *RP11-104J23*.*1* was significantly downregulated (*P* = 0.01) with a higher FC of –5.6 compared with VEH/SIV versus controls. When comparing VEH/SIV with the THC/SIV group, *CCL15* was significantly upregulated (FC = 2.6) (*P* = 0.005) in VEH/SIV RMs. *ITGA6*, an integrin that functions as a cell adhesion and signaling molecule, was upregulated in both THC/SIV (FC = 2.0) and VEH/SIV (FC = 2.9) RMs, while its corresponding lncRNA (*AC078883*.*4*) was downregulated (FCs = 6.8 and 5.9, respectively).

Similarly, significantly decreased expression of 8 protein-coding genes (Supplemental Table 8) was associated with significant upregulation of their respective natural antisense lncRNAs in CE of THC/SIV RMs that also included *MMP25-AS1*, a natural antisense lncRNA that shared significant sequence complementarity to its corresponding protein-coding gene matrix metalloproteinase 25 (*MMP25*). Overall, THC-mediated attenuation of inflammatory responses in CE of chronically SIV-infected RMs involves differential regulation of lncRNA expression.

### Genes associated with pro-inflammatory signaling, IFN response, and antimicrobial defense are significantly upregulated in CE in chronic HIV/SIV infection.

**Figure 2:**
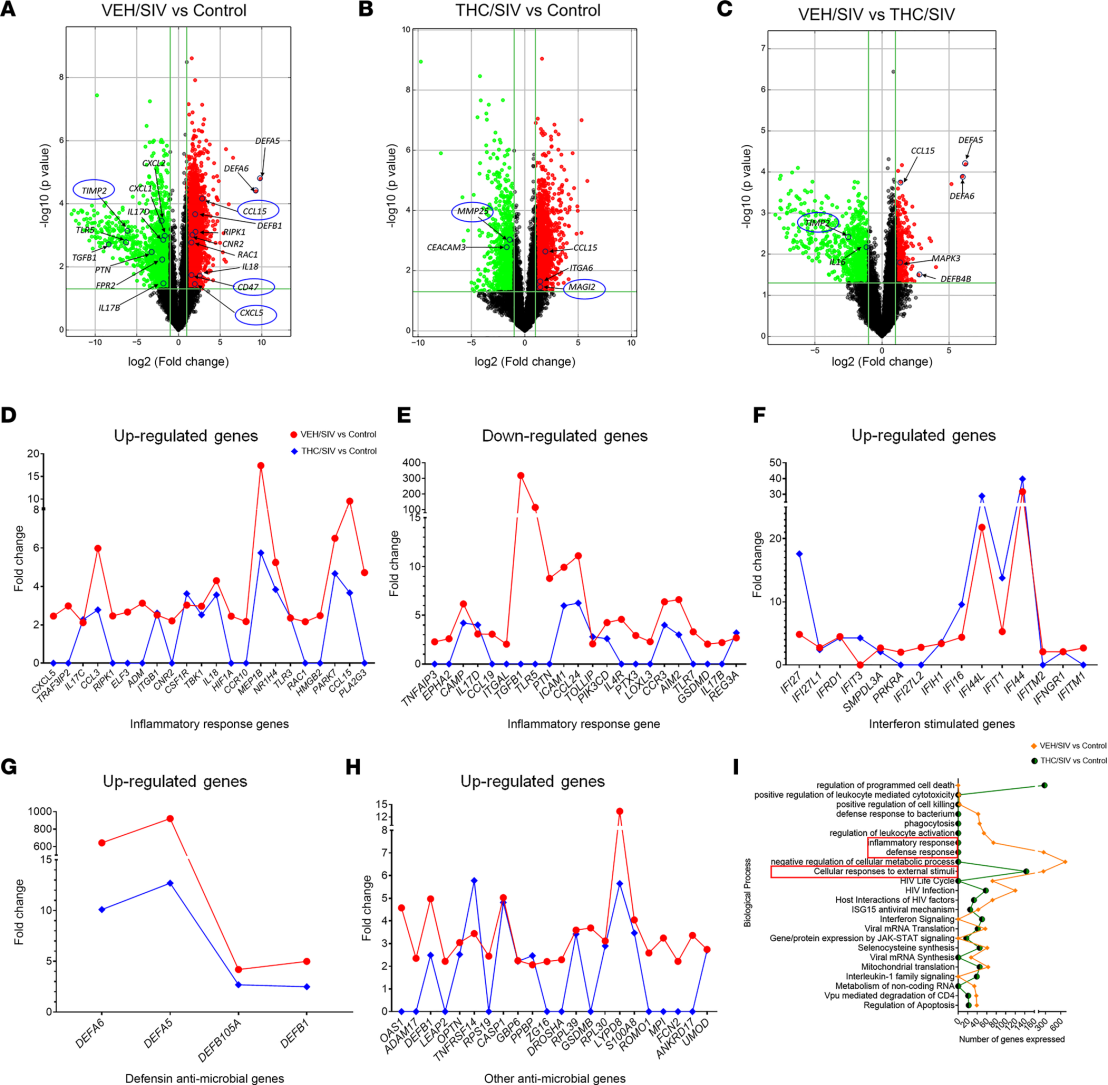
Impact of THC on pro-inflammatory and antimicrobial defense genes in CE of SIV-infected RMs. Volcano plots show the relationship between fold-change (*x* axis) and statistical significance (*y* axis) of DE mRNAs in the CE of VEH/SIV (*n* = 5) (**A**) and THC/SIV (*n* = 6) (**B**) relative to control (*n* = 6) RMs and VEH/SIV relative to THC/SIV RMs (**C**). The negative log of statistical significance (*P* value) (base 10) is plotted on the *y* axis, and the log of the fold-change (base 2) is plotted on the *x* axis. Notable DE mRNAs are shown in the volcano plots. Significantly upregulated (**D**) and downregulated (**E**) DE inflammation-associated genes in CE of VEH/SIV (shown in red) and THC/SIV (blue) RMs. Significantly upregulated IFN-stimulated genes (**F**), defensins (**G**), and other antimicrobial genes (**H**) in CE of VEH/SIV (red) and THC/SIV (blue). GO enrichment analysis of DE protein-coding genes in VEH/SIV (orange) and THC/SIV (green) relative to control RMs that grouped into the different functional clusters/biological processes (**I**).

In addition to identifying DE lncRNAs, the microarray platform enabled the profiling of mRNAs. As evident in Figure 2, A and B, relative to uninfected controls, a greater number of differentially upregulated mRNAs were identified in CE of VEH/SIV (*n* = 4,904) compared with THC/SIV (*n* = 3,065) RMs. Similarly, the number of significantly downregulated mRNAs also showed a similar trend with a higher number of mRNAs showing significantly reduced expression in CE of VEH/SIV (*n* = 1,629) compared with THC/SIV (*n* = 1,164) RMs. When comparing the VEH/SIV and THC/SIV (Figure 2C) groups, 555 and 719 mRNAs showed significantly increased and decreased expression, respectively. Additionally, significantly higher numbers of DE inflammation-associated genes were detected in CE of VEH/SIV compared with THC/SIV RMs (Figure 2D). In terms of FC, the expression of more than 81% of the upregulated pro-inflammatory genes shown in Figure 2D was significantly decreased or not DE in CE of THC/SIV RMs. As evident in Figure 2D, the expression of several pro-inflammatory genes such as *CXCL5*, *RIPK1*, *ELF3*, *ADM*, *CNR2*, *TBK1*, *IL18*, *MEP1β*, *RAC1*, *PARK7*, and *PLA2G3* was significantly higher in CE of VEH/SIV but not THC/SIV RMs. Further, the expression of a subset of genes associated with intestinal epithelial homeostasis such as *TGFβ1*, *TLR5*, *PTN*, *PTX3*, and *IL17B* was significantly downregulated in CE of VEH/SIV RMs (Figure 2E). Most strikingly, 59% of the genes that included *TGFβ1*, *TLR5*, *PTN*, *PTX3*, and *IL17B* shown in Figure 2E were not perturbed in CE of THC/SIV RMs. Further, relative to uninfected controls, the expression of genes linked to IFN response (Figure 2F), epithelial defense (defensins) (Figure 2G), and antimicrobial functions (Figure 2H) was significantly elevated in the CE of VEH/SIV compared with the THC/SIV group.

Gene Ontology (GO) enrichment analysis of DE protein-coding genes identified in VEH/SIV and THC/SIV relative to uninfected controls demonstrated significant differences in the numbers of total genes that grouped into the different functional clusters/biological processes (Figure 2I). Gene enrichment analysis showed differential enrichment of genes in VEH/SIV RMs for biological functions involved in inflammatory response (*n* = 74), immune defense response (*n* = 261), and negative regulation of cellular metabolic processes (*n* = 688). Interestingly, none of the DE genes in CE of THC/SIV RMs clustered in any of the 3 inflammation-associated biological processes. The CE of the THC/SIV group expressed 143 genes that grouped in the “cellular response to external stimuli” cluster, while the VEH/SIV group had 257 genes that clustered in this group. These findings underscore the intestinal epithelial protective properties of low-dose phytocannabinoids during chronic HIV/SIV infection even in the absence of cART.

### Protein expression of MMP25 (MT6-MMP) is significantly elevated in colon of VEH/SIV RMs and localized to the CE and lamina propria mononuclear cells.

**Figure 3:**
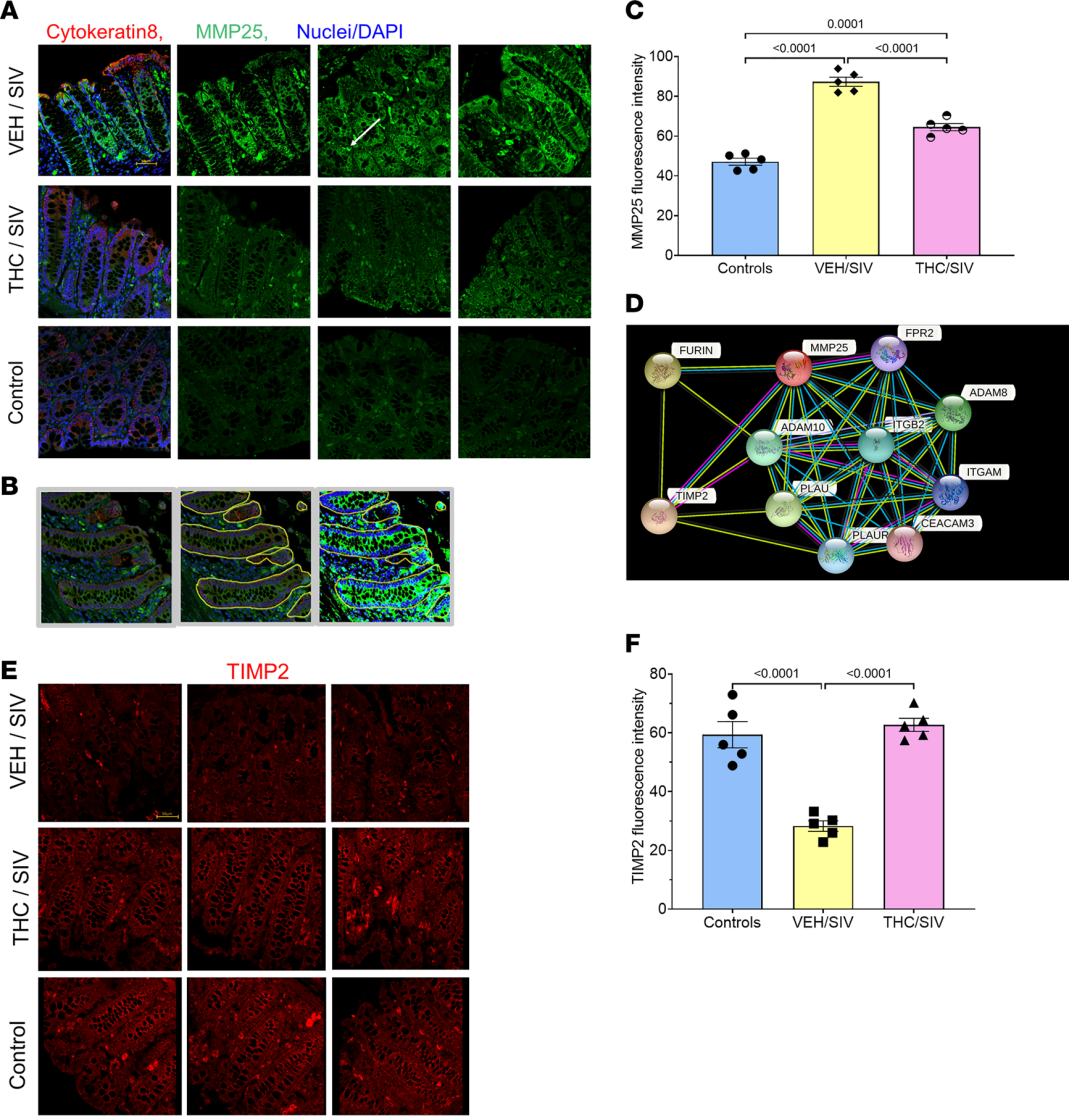
THC decreases MMP25 while elevating TIMP2 protein expression in CE of SIV-infected RMs. Whole colon tissue sections were immunostained for MMP25 (**A**) (green) and TIMP2 (**E**) (red) and DAPI for nuclear staining (blue). Note the increased MMP25 (**A**) and decreased TIMP2 (**E**) protein expression, specifically in CE of VEH/SIV (*n* = 5) compared with THC/SIV (*n* = 5) and control (*n* = 5) RMs. Representative immunofluorescence images were captured using a Zeiss confocal microscope at 20× original magnification. Scale bars: 50 μm. The demarcation of CE regions excluding the lamina propria compartment using the freehand tool in HALO (**B**). Differences in MMP25 (**C**) and TIMP2 (**F**) signal intensity between groups were analyzed using 1-way ANOVA employing the Prism v9 (GraphPad Prism). A *P* value of <0.05 was considered significant. Data represent mean ± SEM. STRING v 11.0 software was used to predict the MMP25 protein-protein interactions with other proteins (**D**). Each node represents all the proteins produced by a single protein-coding gene locus. Edges represent protein-protein associations.

We further characterized MMP25 protein expression levels in the colon for a couple of reasons. First, MMP25 is a plasma membrane-type MMP (MT-MMP) (binds to plasma membrane via a glycosylphosphatidylinositol anchor) that plays an important role in the innate immune response and proinflammatory cytokine production (29, 30). Second, it promotes inflammation by inactivating alpha-1 proteinase inhibitor (alpha-1 anti-trypsin/SERPINA1) (31), a major tissue protectant against proteolytic enzymes released by activated neutrophils, thereby facilitating transendothelial migration of neutrophils into the intestinal lamina propria and thereafter transepithelial migration. The latter event can result in secretory diarrhea, epithelial barrier disruption, and microbial translocation, 3 critical pathogenic events associated with HIV/SIV enteropathy. As seen in Figure 3A, MMP25 protein expression localized to cytokeratin 8–positive (red staining) CE and –negative lamina propria cells. Since our lncRNA microarray profiling was performed using purified CE without lamina propria cells, we quantified MMP25 expression exclusively in CE by marking epithelial regions using the freehand tool available in HALO (Figure 3B). In agreement with microarray data, MMP25 staining intensity (green) was weaker in CE of THC/SIV and uninfected control RMs (Figure 3A). Overall, MMP25 protein expression was elevated in CE of VEH/SIV RMs. Image quantification (HALO) confirmed significantly elevated MMP25 protein expression in the CE of VEH/SIV compared with both THC/SIV (*P* < 0.0001) and uninfected control RMs (~2.0-fold) (*P* < 0.0001) (Figure 3C). MMP25 protein expression was also significantly elevated in CE of THC/SIV compared with uninfected control RMs (*P* = 0.0001), however, by less than 1.5-fold (Figure 3C).

STRING v 11.0 software was used to predict potential interactions of MMP25 protein with other proteins (Figure 3D) that included both direct physical and indirect functional associations between proteins. This analysis found MMP25 protein to directly associate with CEACAM3, TIMP2, a disintegrin and a metalloproteinase domain-containing protein 10 (ADAM10), ADAM8, FURIN, formyl peptide receptor 2 (FPR2), PLAU, PLAUR, ITGB2, and ITGAM proteins. Interestingly, in our microarray data, *TIMP2* (FC = 102.34) (*P* = 0.0005), a negative regulator of MMP25 protein function (32), and FPR2 (FC = 2.3) (*P* = 0.006), an important component of the innate immune response against bacterial infections (33), were significantly downregulated in CE of VEH/SIV compared with uninfected controls. *ADAM10* was significantly upregulated in CE of both VEH/SIV (FC = 3.1) (*P* = 0.0017) and THC/SIV (FC = 2.6) (*P* = 0.005) RMs, while *ADAM8* was significantly downregulated in both groups (VEH/SIV, FC = 9.0, *P* < 0.0001; and THC/SIV, FC = 3.1, *P* = 0.002).

Given that TIMP2 is a negative regulator of MMP25 activity and since our microarray data showed significant downregulation of TIMP2 (102-fold) only in VEH/SIV but not in THC/SIV RMs, we next determined if the same expression pattern existed at the protein level in CE. Interestingly, in agreement with the microarray data, TIMP2 protein (red) expression was significantly downregulated in CE of VEH/SIV relative to controls (*P* < 0.0001) and THC/SIV RMs (*P* < 0.0001) (Figure 3, E and F). In contrast, TIMP2 protein expression levels in CE of THC/SIV were no different from uninfected control RMs (Figure 3F). Collectively, high TIMP2 protein expression (Figure 3F) was associated with moderate MMP25 protein expression levels (Figure 3C) in CE of THC/SIV RMs. Intriguingly, the opposite expression pattern was detected in CE of VEH/SIV RMs (Figure 3, C and F).

### THC reciprocally regulates the expression of the natural antisense lncRNA MMP25-AS1 and its associated protein-coding gene MMP25.

**Figure 4:**
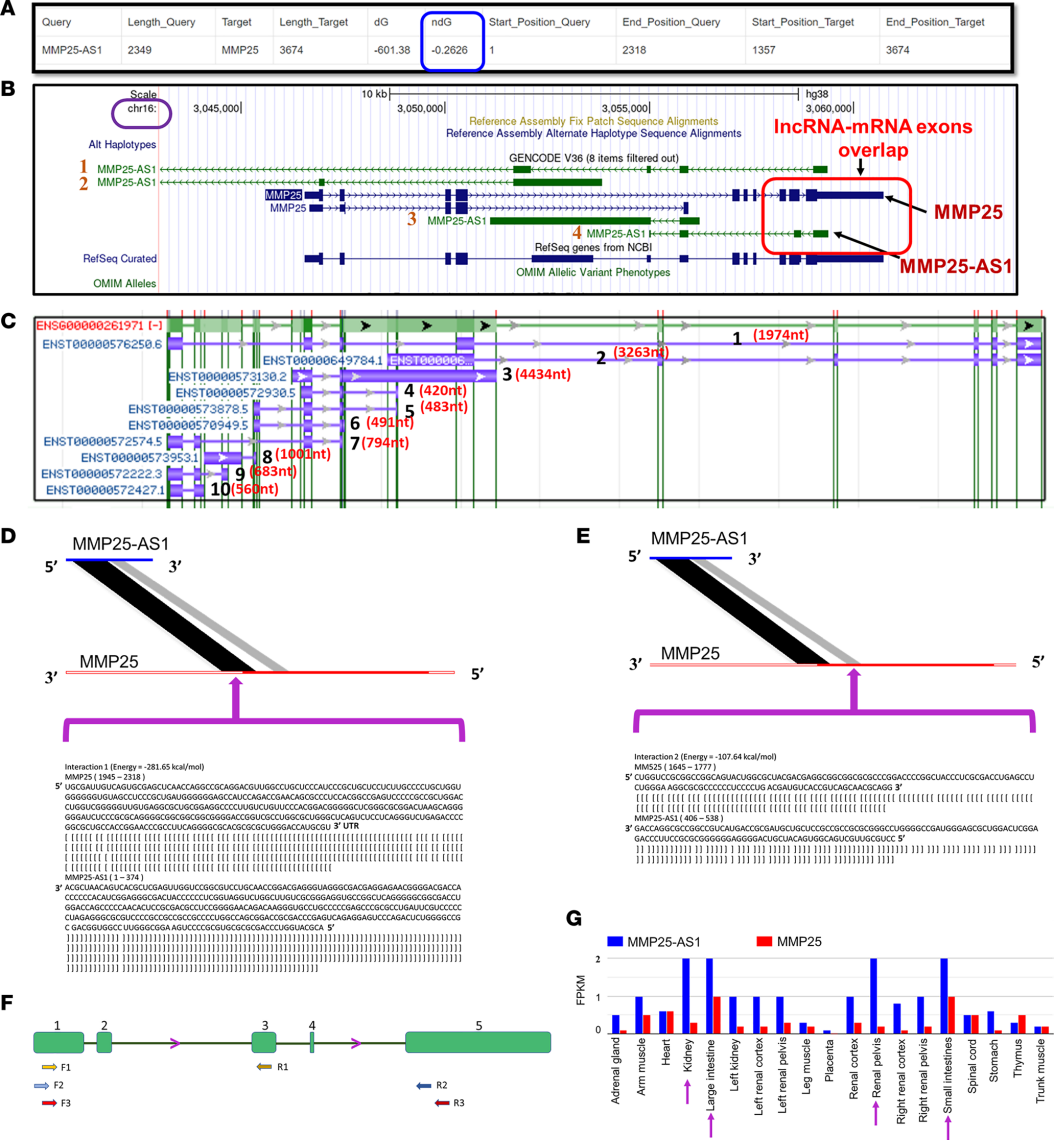
Computational predictions of interactions between *MMP25-AS1* and its associated protein-coding gene *MMP25*. LncTar software predictions of the normalized binding free energy (ndG) for the complementary regions between *MMP25-AS1* and *MMP25* (**A**). Note the minimum ndG (blue box) is –0.2626. Screenshot taken from the UCSC Genome Browser shows the genomic locations of *MMP25* mRNA, *MMP25-AS1* isoforms, and the 3′UTR genomic overlap (red circle) between exons 1 and 2 of the *MMP25-AS1* isoform 4 (794 bp) and exons 9 and 10 of *MMP25* mRNA (**B**). Screenshot of the NCBI Gene database page showing all 10 isoforms of *MMP25-AS1* and their sizes (**C**). The sizes represent the total exon length of each isoform. CLUSTALW sequence alignment of *MMP25-AS1/MMP25* showing complementary interactions (**D** and **E**). Note the presence of a large (**D**) (374 bp) and small perfect complementary region (**E**) (133 bp) between *MMP25* and *MMP25-AS1*. Representation of exonic regions (regions 1–5) on the *MMP25-AS1* isoform ENST00000572574.5 (794 bp) and the binding sites of the 3 PCR primer pairs used to amplify the RM 794 bp isoform (**F**). Expression patterns of *MMP25-AS1* and *MMP25* in 19 different tissues obtained from fetuses with congenital diseases (**G**) (data obtained from NIH epigenomics program). FPKM, Fragment per kilobase of transcript per million fragments mapped.

The significantly decreased *MMP25* mRNA (FC = 2.2) and elevated expression of its natural antisense lncRNA, *MMP25-AS1* (FC = 2.3), suggested the possibility for epigenetic regulation, and therefore, we hypothesized potential interactions between *MMP25* and *MMP25-AS1*. Accordingly, we used CLUSTALW and LncTar software (34) to determine if regions of complementarity existed, predict the minimum free energy, and calculate the normalized binding free energy (ndG) between the full-length *MMP25-AS1* transcript (2,349 bp) and *MMP25* mRNA. In LncTar, the cutoff value for the ndG was set to the recommended value of –0.1. The ndG for the *MMP25-AS1*/*MMP25* mRNA interaction was predicted to be –0.2626, verifying an energetically stable duplex structure (blue square in Figure 4A). However, multiple isoforms of *MMP25-AS1* (UCSC Genome Browser and National Center for Biotechnology Information [NCBI] Gene database) have been identified, and the genomic locations of these isoforms are shown in Figure 4, B and C. As evident in the red-circled region in Figure 4B, the *MMP25-AS1* isoform 4 (ENST00000572574.5) has 2 regions of sequence similarity with *MMP25* mRNA (NM_022468.5). Note that lncRNA *MMP25-AS1* is located on chromosome 16, and its 5′ end sequence overlaps with the 3′UTR of *MMP25* mRNA. Further analysis of *MMP25-AS1*/*MMP25* mRNA sequence alignment using CLUSTALW identified 2 regions (large region, 374 bp, and small region, 133 bp) with perfect complementarity between corresponding exonic 1 and 2 regions of the *MMP25-AS1* isoform ENST00000572574.5 (isoform 4 in Figure 4B and isoform 7 in Figure 4C) with exon regions 10 (Figure 4, B and D, and Supplemental Figure 1A) and 9 (Figure 4, B and E, and Supplemental Figure 1A) of *MMP25*, respectively, suggesting the potential for both transcriptional and functional regulation of *MMP25* by *MMP25-AS1*. Note the low free energy values of –281.65 and –107.64 kcal/mol for both large and small complementary region interactions, respectively (Figure 4, D and E). Since there are multiple isoforms of *MMP25-AS1*, we next determined if isoform ENST00000572574.5 (794 bp), which has the 2 complementary regions with *MMP25*, was expressed in the intestinal epithelium of RMs. As shown in Figure 4F, only the primer pair that spanned exonic regions 1 and 3 of *MMP25-AS1* successfully amplified a PCR product that had perfect homology (Supplemental Figure 2A) with the 794 bp isoform, which includes the 2 regions that shared perfect complementarity with *MMP25* mRNA, thus verifying the presence of the 794 bp isoform in CE of RMs. Note that exons 1 and 3 are present only in isoform ENST00000572574.5 (isoform 7 in Figure 4C). Therefore, we can verify that the amplified PCR product was generated solely from isoform ENST00000572574.5. Interestingly, CLUSTALW alignment confirmed significant complementarity between the human *MMP25-AS1* isoform and *Macaca mulatta* (RM) predicted *MMP25* transcript variant-2 (Supplemental Figure 2B). More importantly, the amplified RM *MMP25-AS1* sequence shared 99% homology with the human *MMP25-AS1* isoform ENST00000572574.5 (Supplemental Figure 2C). Based on the NIH epigenomics program data, the expression patterns of *MMP25-AS1* and *MMP25* in 19 tissues obtained from fetuses with congenital diseases are shown in Figure 4G. Note that with the exception of the heart, spinal cord, placenta, thymus, and trunk muscles, expression levels of *MMP25* are considerably lower than that of *MMP25-AS1* in the 14 other tissues examined. The predominant baseline expression pattern showed decreased *MMP25* and increased *MMP25-AS1* expression in 14 out of 19 tissues examined. Interestingly, among all tissues, *MMP25-AS1* expression was highest in both small and large intestine and kidney (pelvis). Also, note that *MMP25* mRNA expression was highest in both small and large intestine compared with other organs (Figure 4G).

### MMP25-AS1 overexpression significantly reduces MMP25 mRNA and protein expression in in vitro–cultured primary colon and small intestinal epithelial cells.

**Figure 5:**
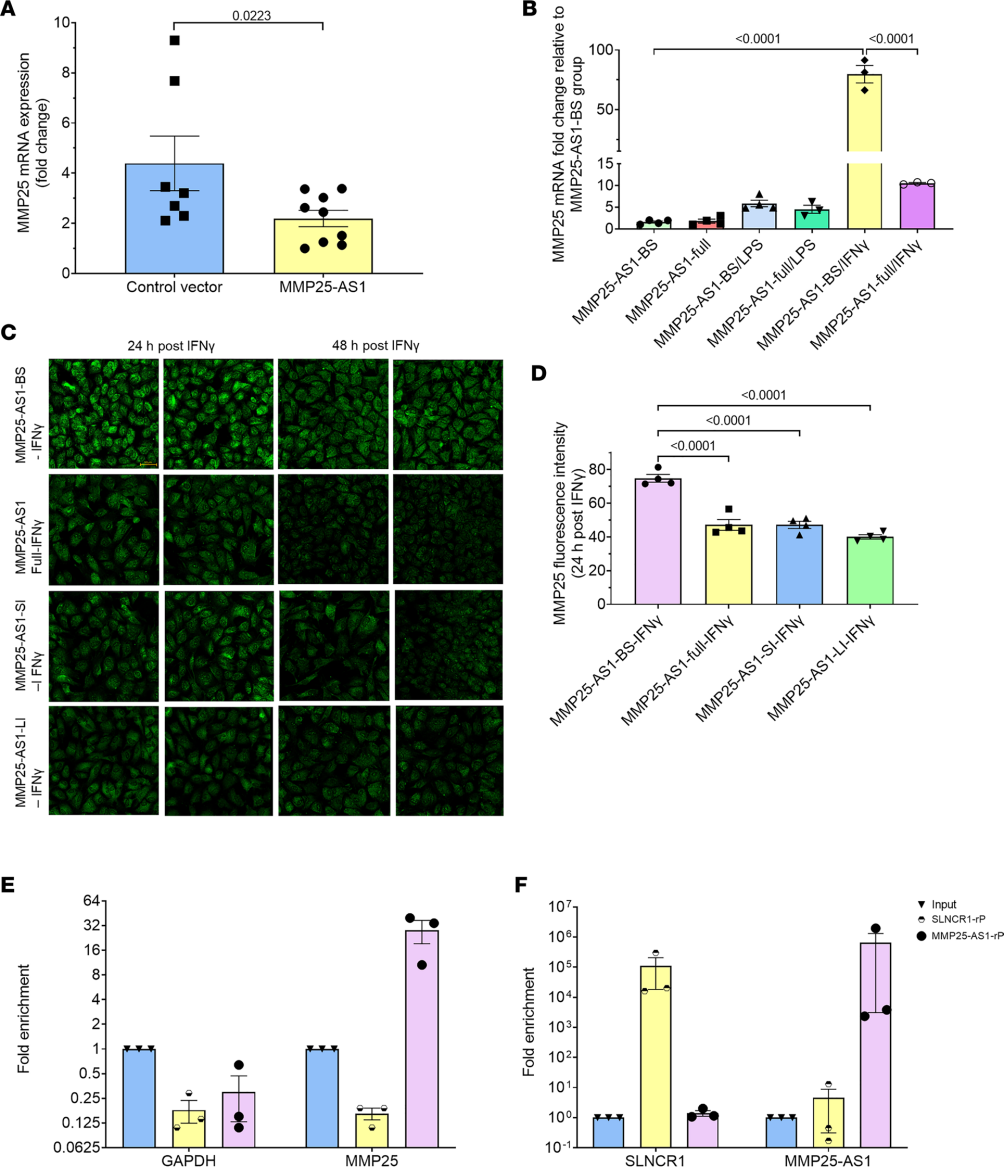
*MMP25-AS1* overexpression significantly reduces MMP25 mRNA/protein expression in primary human colonic epithelial and human small intestinal epithelial cells in vitro. Reverse transcription quantitative PCR (RT-qPCR) quantitation of *MMP25* mRNA expression levels in human colonic epithelial (hCE) cells overexpressing *MMP25-AS1* (*n* = 9) or control vector (*n* = 7) (**A**). *MMP25* mRNA expression in human small intestinal epithelial (hSIE) cells overexpressing *MMP25-AS1*-BS or *MMP25-AS1*-full length RNA following stimulation with LPS or IFN-γ (**B**). One-way ANOVA (GraphPad Prism) was used to analyze ΔC_T_ values. A *P* value of <0.05 was considered significant. MMP25 protein expression in hSIE cells overexpressing *MMP25-AS1*-BS (*n* = 4) or *MMP25-AS1*-full (*n* = 4) or *MMP25-AS1*-SI (*n* = 4) or *MMP25-AS1*-LI (*n* = 4) in response to IFN-γ treatment (24 hours and 48 hours after IFN-γ treatment) (**C**). Immunofluorescence images were captured using a Zeiss confocal microscope at 20× original magnification. MMP25 signal intensity was quantified using HALO (highplex FL method). Differences in MMP25 signal intensity between treatment groups were analyzed using 1-way ANOVA employing the Prism v9 (GraphPad Prism) (**D**). A *P* value of <0.05 was considered significant. RT-qPCR quantitation of *MMP25* mRNA levels following pull-down using biotinylated full-length *MMP25-AS1* or control *SLNCR1* RNA incubated with hSIE cellular lysates (**E**). RT-qPCR confirmation of the presence of sufficient amounts of biotinylated full-length *MMP25-AS1* or control *SLNCR1* RNA in hSIE cellular lysates (**F**). Data represent mean ± SEM. *MMP25-AS1*-BS, both complementary regions scrambled; *MMP25-AS1*-full, both complementary regions intact; *MMP25-AS1*-SI, small (133 bp complementary region intact); *MMP25-AS1*-LI, large (374 bp complementary region intact).

We next investigated whether MMP25-AS1 acted as a negative regulator/inhibitor of *MMP25* expression in the intestinal epithelium. First, to determine if *MMP25-AS1* acted at the transcriptional level, we quantified *MMP25* mRNA levels in human colonic epithelial (hCE) cells following overexpression of the full-length (~2,349 bp) *MMP25-AS1* lncRNA (*MMP25-AS1*-full). As predicted, *MMP25* mRNA levels were significantly reduced (*P* = 0.0223) in hCE cells that overexpressed *MMP25-AS1* compared with the control vector–transfected (empty pcDNA3.1 vector) cells (Figure 5A). Shapiro-Wilk test confirmed data normality. Due to growth problems with the hCE cells, we pursued further *MMP25-AS1* overexpression experiments using primary human small intestinal epithelial (hSIE) cells that continued to proliferate and grow well. Here, we used a control vector expressing the full-length *MMP25-AS1* sequence with both complementary regions scrambled (*MMP25-AS1*-BS). Unlike hCE cells, hSIE cells did not show significant reduction of baseline *MMP25* mRNA levels in response to *MMP25-AS1*-full overexpression compared with the control vector–transfected (*MMP25-AS1*-BS) cells (Figure 5B).

Next, we determined if inflammatory stimuli like IFN-γ or LPS can induce MMP25 upregulation and whether *MMP25-AS1* can counteract IFN-γ– or LPS-mediated upregulation of *MMP25* mRNA expression. Interestingly, hSIE cells overexpressing *MMP25*-*AS1*-full and subsequently treated with IFN-γ (50 units for 24 hours) showed no significant change in *MMP25* mRNA expression levels compared to untreated cells (Figure 5B). However, hSIE cells transfected with the control vector and subsequently treated with IFN-γ expressed significantly elevated (*P* < 0.0001) *MMP25* mRNA levels compared with untreated cells (Figure 5B). More importantly, control vector–transfected and IFN-γ–treated hSIE cells showed significantly (*P* < 0.0001) higher levels of *MMP25* mRNA expression compared with *MMP25-AS1*-full transfected and IFN-γ–treated cells, attesting that *MMP25-AS1* can successfully block IFN-γ–mediated induction of *MMP25* expression. Treatment of *MMP25-AS1*-full–transfected hSIE cells with LPS did not change *MMP25* mRNA levels significantly at 96 hours compared with control vector–transfected cells (Figure 5B).

After confirming the ability of *MMP25-AS1* to inhibit *MMP25* mRNA expression, we next investigated if these effects extended to the protein level. Furthermore, since there are 2 complementary regions (large and small), we were intrigued to determine if either region differed in its ability to suppress MMP25 protein expression. In the absence of IFN-γ stimulation, hSIE cells overexpressing *MMP25-AS1*-full did not show significant changes in MMP25 protein expression compared with cells transfected with the control vector (*MMP25-AS1*-BS) at 96 hours (data not shown). However, following IFN-γ treatment, hSIE cells transfected with *MMP25-AS1*-BS showed a significant increase in MMP25 protein expression compared with IFN-γ–treated *MMP25-AS1*-full–transfected cells (*P* < 0.0001) (Figure 5, C and D). Cells transfected with the *MMP25-AS1* vector expressing only the intact small complementary region (*MMP25-AS1*-SI) reduced MMP25 protein expression to levels similar to those detected in cells transfected with the *MMP25-AS1*-full vector (Figure 5, C and D). Interestingly, although transfection of cells with *MMP25-AS1*-LI (only large complementary region intact) resulted in slightly greater reduction in MMP25 protein levels as detected with *MMP25-AS1*-SI vector–transfected cells (Figure 5, C and D), this difference was not statistically significant. It is clear from Figure 5, C and D, that the maximum *MMP25-AS1*–mediated reduction in MMP25 protein levels was detected at 24 hours after IFN-γ treatment. Interestingly, reductions in MMP25 protein expression were still detected at 48 hours and 72 hours after IFN-γ treatment of *MMP25-AS1*-full relative to *MMP25-AS1*-BS–transfected cells (Figure 5C and Supplemental Figure 3, A–C). Nevertheless, statistically significant reductions in MMP25 protein were detected only at 48 hours (*P* = 0.0014) (Supplemental Figure 3B) but not 72 hours after IFN-γ treatment (Supplemental Figure 3C). Similarly, cells transfected with *MMP25-AS1*-SI and *MMP25-S1*-LI vectors showed maximum reduction in MMP25 expression at 24 hours followed by further reduction at 48 hours and 72 hours after IFN-γ treatment (Figure 5C and Supplemental Figure 3A). Similar to *MMP25-AS1*-full/IFN-γ–treated cells, both *MMP25-AS1*-SI/IFN-γ– and *MMP25-AS1*-LI/IFN-γ–treated cells showed significant reduction in MMP25 protein expression (*P* < 0.0001 and 0.002, respectively) at 48 hours (Supplemental Figure 3B) compared with *MMP25-AS1*-BS/IFN-γ–treated cells. However, for both vectors, no statistically significant reduction in MMP25 protein levels was observed at 72 hours after IFN-γ treatment compared to *MMP25-AS1*-BS/IFN-γ–treated cells (Supplemental Figure 3C).

### MMP25-AS1 can physically interact with MMP25 through direct binding to complementary regions in exons 1 and 2.

While the presence of 2 (small 133 bp and large 374 bp) perfectly complementary regions may partly explain the reduction in *MMP25* mRNA and protein levels in response to *MMP25-AS1* overexpression, the data do not provide evidence of direct physical interactions between the two. To address this question, we performed RNA pull-down experiments by incubating in vitro synthesized full-length *MMP25-AS1* or a negative control *SLNCR1* with cellular lysates obtained from hSIE cells (unstimulated). As shown in Figure 5, E and F, RNA pull-down using biotin-labeled lncRNA *MMP25-AS1* resulted in an approximately 32-fold enrichment in *MMP25* mRNA compared with the RNA pulled down using the control biotin-labeled *SLNCR1* (Figure 5E). The detection of both labeled *MMP25-AS1* and *SLNCR1* in the lysate (Figure 5F) suggests the presence of both labeled RNAs in the lysate at sufficiently high levels.

### Elevated MMP25 protein expression in colon is associated with increased numbers of CD11b/MPO^++^ neutrophils and enhanced epithelial expression of CD47.

**Figure 6:**
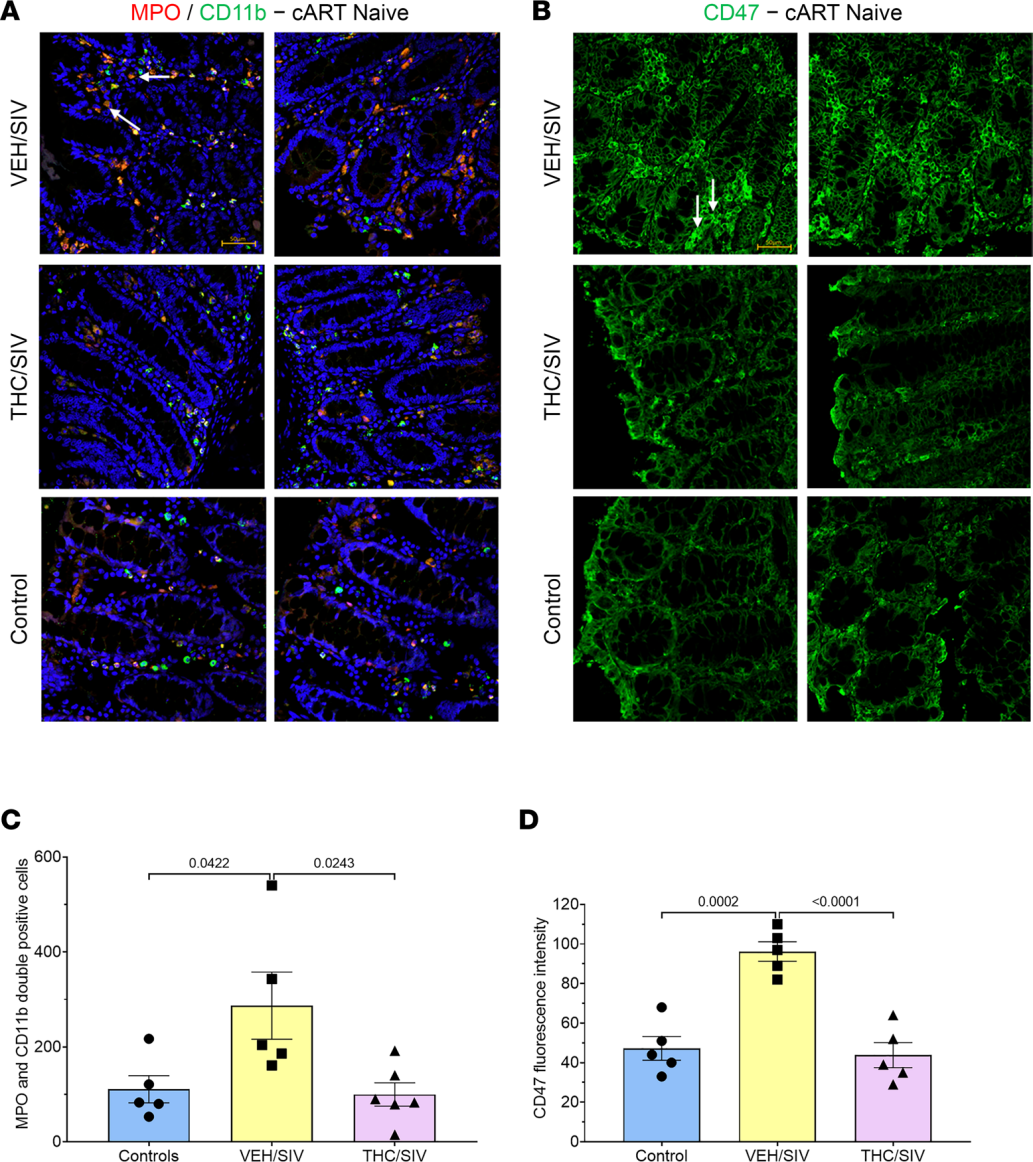
Effect of THC on MPO/CD11b^++^ neutrophil counts and CD47 expression in colon of SIV-infected RMs. Whole colon tissue sections were immunostained with antibodies against MPO (red) and CD11b (green) and DAPI (blue) for nuclear staining (**A**). Note the increased number of MPO/CD11b^++^ neutrophils (yellow) in the colonic lamina propria of VEH/SIV (*n* = 5) relative to THC/SIV (*n* = 6) and control (*n* = 5) RMs. Increased CD47 protein expression (**B**) in CE of VEH/SIV (*n* = 5) relative to THC/SIV (*n* = 5) and control (*n* = 5) RMs. Representative immunofluorescence images were captured using a Zeiss confocal microscope at 20× original magnification. Scale bars: 50 μm. Quantitation of MPO/CD11b^++^ cells was performed using highplex FL method available in HALO. Quantitation of CD47 signal intensity in the CE excluding lamina propria cells was performed using a freehand tool of HALO (area quantification method). Differences in the number of MPO/CD11b^++^ cells (**C**) and CD47 (**D**) signal intensity between groups were analyzed using 1-way ANOVA employing the Prism v9 (GraphPad Prism). A *P* value of <0.05 was considered significant. Data represent mean ± SEM.

Consistent with the neutrophil chemoattractant properties of MMP25, we detected significantly elevated numbers of CD11b/MPO^++^ neutrophils in the colonic lamina propria of VEH/SIV compared with both control (*P* = 0.0422) and THC/SIV RMs (*P* = 0.0243) (Figure 6, A and C). However, no significant changes in the numbers of CD11b/MPO^++^ neutrophils were detected in the colonic lamina propria of THC/SIV compared to control RMs.

Since infiltrated neutrophils in the colonic lamina propria are known to further migrate across CE (neutrophil transepithelial migration) and their rate of migration along the epithelial paracellular space has been shown to be regulated by the epithelial transmembrane glycoprotein CD47, which binds to leukocyte signal regulatory protein-α (35), we next quantified protein expression of CD47 that was significantly increased at the mRNA level in CE of VEH/SIV (Figure 2A) but not THC/SIV relative to control RMs. In agreement with the gene expression data, we validated significantly high CD47 protein expression exclusively in the CE of VEH/SIV but not THC/SIV RMs compared with control RMs (*P* = 0.0002) (Figure 6, B and D). Interestingly, VEH/SIV RMs also showed significantly elevated (*P* < 0.0001) CD47 protein expression compared with THC/SIV RMs.

### MMP25 expression is significantly elevated in jejunum of cART-experienced SIV-infected RMs while MMP25-AS1 expression is significantly decreased.

**Figure 7:**
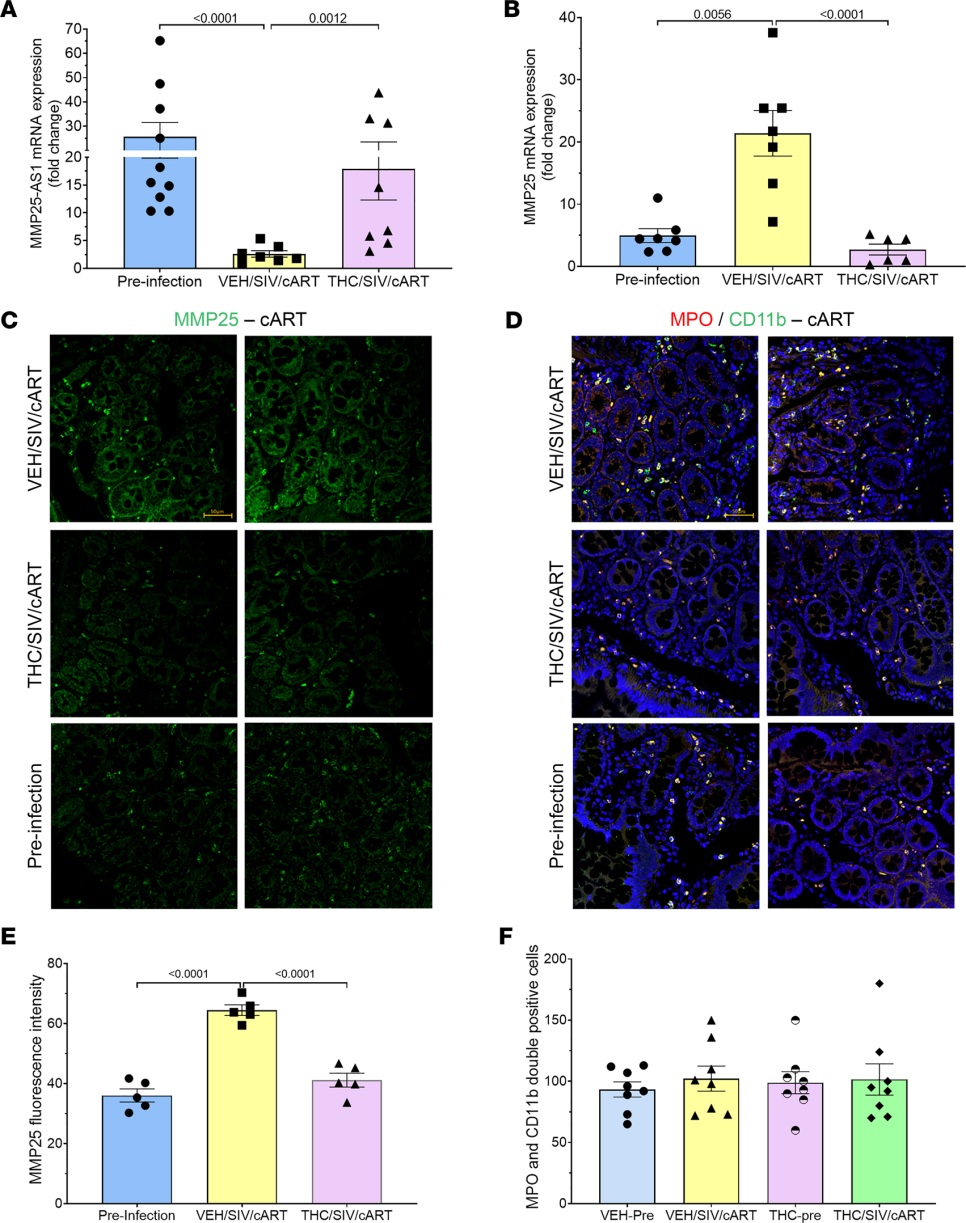
Effect of THC and cART on *MMP25-AS1* and *MMP25* expression in JE of SIV-infected RMs. RT-qPCR quantitation of *MMP25-AS1* lncRNA expression (**A**) and *MMP25* mRNA expression (**B**) levels in VEH/SIV/cART (*n* = 8) and THC/SIV/cART (*n* = 8) RMs before (Pre-infection) (*n* = 10) and at 5 months after SIV infection. One-way ANOVA (GraphPad Prism) was used to analyze ΔC_T_ values. A *P* value of <0.05 was considered significant. MMP25 protein expression in JE of VEH/SIV/cART (*n* = 5) and THC/SIV/cART (*n* = 5) RMs before (Pre-infection) (*n* = 5) and at 5 months after SIV infection (**C**). Whole jejunum tissue sections were immunostained with antibodies against MMP25 (green). Note the increased MMP25 expression in JE of VEH/SIV/cART compared with THC/SIV/cART and the preinfection time point. Whole jejunum tissue sections from VEH/SIV/cART-5MPI (*n* = 8) and VEH/preinfection (*n* = 8), and THC/SIV/cART-5MPI (*n* = 8) and THC/preinfection (*n* = 8), were immunostained with antibodies against MPO (red) and CD11b (green), and DAPI (blue) for nuclear staining (**D**). Note the presence of MPO/CD11b^++^ neutrophils (yellow) in the jejunum lamina propria. Representative immunofluorescence images were captured using a Zeiss confocal microscope at 20× original magnification. Scale bars: 50 μm. Quantifying MMP25 signal intensity, specifically in the JE excluding lamina propria cells, was performed using a freehand tool available in HALO (area quantification method). MPO/CD11b^++^ cells were quantified using the highplex FL method available in HALO. Differences in MMP25 signal intensity (**E**) and MPO/CD11b^++^ cell numbers (**F**) between groups were analyzed using 1-way ANOVA employing the Prism v9 (GraphPad Prism). A *P* value of <0.05 was considered significant. Data represent mean ± SEM.

Since intestinal epithelial barrier dysfunction and dysbiosis persist despite viral suppression by cART (3), the significantly higher expression of MMP25 in CE of cART-naive RMs persuaded us to further examine if similar expression patterns existed in cART-treated SIV-infected RMs and whether THC influenced its expression. Therefore, we quantified MMP25 and MMP25-AS1 in jejunal epithelial cells isolated from cART-experienced (5 months’ treatment) RMs for 2 reasons. First, GI inflammation persists in PLWH on cART (3), and unlike other intestinal diseases, HIV affects the entire intestinal tract, which includes both small and large intestine (36). Second, based on the NIH epigenomics program data (Figure 4G), MMP25-AS1 expression was equally high in both small and large intestine. Consequently, the next logical step was to determine if similar HIV/SIV-induced perturbations in MMP25 expression detected in the colon also occurred in the small intestine (jejunum). Accordingly, we first quantified *MMP25-AS1* RNA in jejunum epithelial cell RNA isolated from jejunal surgical resections obtained from VEH/SIV/cART and THC/SIV/cART at 5 MPI and the preinfection time point (controls) using primers that amplified the *MMP25-AS1* isoform ENST00000572574.5. Interestingly, we not only verified the presence of *MMP25-AS1* isoform ENST00000572574.5 in the jejunal epithelium (JE) but also detected significantly decreased expression of the *MMP25-AS1* isoform in VEH/SIV/cART compared with THC/SIV/cART (*P* = 0.0012) and preinfection control RMs (*P* < 0.0001) (Figure 7A). *MMP25-AS1* expression in JE of THC/SIV/cART RMs did not differ from preinfection time point. Further, relative to VEH/SIV/cART RMs, expression of *MMP25-AS1* was significantly higher in JE of THC/SIV/cART RMs, and this increase was also associated with significantly reduced *MMP25* mRNA expression (*P* < 0.0001) (Figure 7B). Consistent with increased *MMP25* mRNA expression (Figure 7B), we detected significantly higher MMP25 protein expression in the JE of VEH/SIV/cART compared with THC/SIV/cART (*P* < 0.0001) and preinfection time point (*P* = 0.0001) (Figure 7, C and E). In contrast to the findings in the colon of cART-naive SIV-infected RMs (Figure 6, A and C), no significant difference in the number of MPO/CD11b^++^ neutrophils was detected in the jejunal lamina propria of VEH/SIV/cART compared with THC/SIV/cART RMs despite significantly high MMP25 protein expression. Further, no difference in the number of MPO/CD11b^++^ neutrophils was detected in both groups compared with their respective preinfection samples (Figure 7, D and F). All cART-treated animals showed undetectable plasma and jejunum viral loads at 6 MPI (Supplemental Table 9).

## Discussion

Although the advent of cART has prolonged the life span of PLWH, they have an elevated risk for developing non–AIDS-associated comorbidities, such as cardiovascular disease, HIV-associated neurocognitive disorder, metabolic dysfunction/disease, and so on. Despite immune restoration by cART in the periphery, immune dysfunction persists in the GI tract, leading to dysbiosis, epithelial barrier defects, and translocation of immune-activating microbial products that can drive inflammation-mediated comorbidities (37). While intestinal epithelial permeability defects are a hallmark of HIV/SIV infection and persist in PLWH on cART, the underlying mechanisms remain unclear. Regulation of epithelial barrier function is complex, and though emerging evidence suggests the involvement of epigenetic mechanisms (38) [DNA methylation, histone modifications, and noncoding RNAs (miRNAs and lncRNAs)] and their dysregulation in intestinal diseases like IBD (19), their role in the pathogenesis of HIV/SIV-induced epithelial barrier dysfunction remains unknown and unaddressed.

Noncoding RNAs, though lacking protein-coding capacity, have emerged as critical regulators of gene expression at the transcriptional and posttranscriptional levels in both health and disease (39, 40). Specifically, lncRNAs play important epigenetic regulatory roles in chromatin remodeling, transcriptional control, and posttranscriptional processing and show tissue specific expression (39). Recent findings demonstrated that they may have their own DNA binding motifs, promotor regions, and transcription factor binding sites (41). Further, bioinformatics studies suggest that the expression of protein-coding genes is influenced by lncRNAs, and the transcription of lncRNAs can occur independently of protein-coding genes (41, 42). In IBD, an autoimmune disease associated with recurring intestinal inflammation and epithelial barrier impairment, lncRNAs have been shown to regulate epithelial function and apoptosis, inflammatory response, metabolism, and intercellular communications (21). Accordingly, to address an important knowledge gap in our understanding of HIV/SIV-induced GI epithelial dysfunction, we explored the differential transcriptomic landscape of lncRNAs exclusively in CE of chronically SIV-infected RMs and its modulation by long-term low-dose THC. In the present study, we provide new knowledge on the role of lncRNAs as epigenetic regulators of HIV/SIV-induced intestinal epithelial dysfunction and its modulation by long-term low-dose phytocannabinoids in cART-naive and -experienced SIV-infected RMs.

Relative to controls, greater numbers of lncRNAs were DE in CE of VEH/SIV RMs that included the well-characterized *HOTAIR*, pro-inflammatory *MALAT1* (24), *GATA6-AS1* (22, 43), *GATA3-AS1*, and *SPRY-IT1* (24) that were exclusively upregulated in CE of VEH/SIV RMs. In contrast, fewer lncRNAs were DE in the CE of THC/SIV RMs that comprised *NEAT1*, *IFNG-AS1*, *MMP25-AS1*, and *BISPR* that were significantly upregulated, including *MAGI2-AS3*, with a potential role in regulating epithelial barrier function that showed significantly reduced expression. Interestingly, *IFNG-AS1* was shown to be upregulated in the colon of patients with IBD and to enhance IFN-γ production (44). The finding that *IFNG*-*AS1* knockdown in human T helper cells significantly reduces *IFNγ* gene expression (45) suggests that *IFNG-AS1* is an epigenetic activator of *IFNγ*. In other studies, overexpression of *IFNG-AS1* enhanced *IFNγ* expression, resulting in decreased *Salmonella enterica* typhimurium pathogenesis (46). Similar to these findings, *IFNG-AS1* upregulation was associated with a statistically insignificant (*P* = 0.07) increase (2.6-fold) in *IFNγ* mRNA expression in CE of THC/SIV RMs. Collectively, the selective induction of *IFNG-AS1* may represent an epigenetic mechanism by which THC strengthens the intestinal epithelial antimicrobial defense.

Based on GO analysis, a significantly higher number of DE genes associated with the inflammatory response (*n* = 74 vs. 0), defense response (*n* = 261 vs. 0), negative regulation of cellular metabolic process (*n* = 688), and cellular response to external stimuli (*n* = 257 vs. 143) were detected in CE of VEH/SIV but not THC/SIV RMs relative to uninfected control RMs. The Arraystar human microarray platform allowed the identification of several potentially previously unreported inflammatory response genes in the CE of VEH/SIV RMs with established roles in driving intestinal epithelial dysfunction. The significant upregulation of *CCL15* together with *CXCL5* and *MMP25* in CE of VEH/SIV compared with THC/SIV RMs may partly explain the increased numbers of MPO/CD11b^++^ neutrophils detected in the colonic lamina propria of VEH/SIV RMs. The reduced expression of the majority of these pro-inflammatory genes in CE of THC/SIV RMs provides strong evidence that THC exerts its antiinflammatory/protective effects separately on intestinal epithelial and lamina propria cellular compartments potentially via binding to distinct cannabinoid receptors (CB1R and CB2R). Interestingly, the VEH/SIV group also showed significant downregulation of several genes that have antiinflammatory/antimicrobial (*IL17B*, *IL17D*, *TLR5*, *PTEN*, *FPR2*) (33, 47–50) and epithelial barrier maintenance (*TGFB1*) (51, 52) functions. Moreover, the lack of *FPR2* was associated with selective impairment in the production of chemokines *CXCL1* and *CXCL2* (33). Interestingly, VEH/SIV but not THC/SIV RMs showed significantly reduced expression of all 3 genes, *FPR2* (*P* = 0.006), *CXCL1* (*P* = 0.001) and *CXCL2* (*P* = 0.0002), suggesting that their decreased expression may diminish epithelial antibacterial responses in VEH/SIV RMs. Furthermore, the VEH/SIV group showed significant upregulation of several IFN-stimulated and antimicrobial defensin genes compared with the THC/SIV group, which clearly highlights the antiinflammatory and potentially antidysbiotic properties of THC in the colon of SIV-infected RMs.

Most strikingly, RNA-Seq and immunofluorescence studies confirmed that the elevated MMP25 expression in CE during chronic SIV infection may be greatly facilitated by the significant downregulation of its natural protein inhibitor TIMP2 (Figure 3, E and F). Unlike the positive outcomes with *IFNG-AS1-IFNγ* interactions, upregulation of *MMP25-AS1* was associated with significantly decreased *MMP25* mRNA expression (*P* = 0.0008) in CE of THC/SIV RMs. In contrast to other MMP family members, the *MMP25* protein is a membrane-type MMP that remains attached to the plasma membrane. During viral infection or inflammation, MMP25 inactivates alpha-1 proteinase inhibitor (SERPINA1), the major tissue protectant against proteolytic enzymes released by active neutrophils, thus promoting the migration of neutrophils to sites of active inflammation. Being a natural protein inhibitor of MMP25, the significant downregulation of TIMP2 potentially facilitated the significantly enhanced MMP25 protein expression in CE of VEH/SIV RMs.

While TIMP2 is a well-established protein inhibitor of MMP25 and other MMPs, bioinformatics analysis identified a potential natural antisense lncRNA regulator, namely, *MMP25-AS1*, transcribed from the opposite strand that shared 2 large regions with perfect Watson-Crick complementarity and also showed markedly low binding free energy values, suggesting the potential for direct regulation of *MMP25* by *MMP25-AS1*. Consistent with the presence of these complementary matching regions, overexpression of the full-length *MMP25-AS1* in hCEs significantly reduced baseline *MMP25* mRNA expression, thereby confirming the ability of *MMP25-AS1* to downregulate *MMP25* expression. This represents a previously described lncRNA mechanism involving direct binding to destabilize the mRNA, wherein duplex formation between *7SL* lncRNA and *p53* mRNA prevented binding of the mRNA stabilizing protein, human antigen R (HuR) to p53 mRNA (53). Moreover, HuR- and HuB-dependent stabilization of other MMPs like *MMP9* mRNA has been previously demonstrated in neurons (54, 55). Nevertheless, the lack of change in MMP25 protein expression in hSIE cells prompted us to induce MMP25 protein expression by stimulating the cells with IFN-γ and LPS. Interestingly, while both IFN-γ and LPS activated MMP25 expression in hSIE cells, IFN-γ turned out to be a stronger and significant inducer of MMP25 expression. Further, and more importantly, overexpression of *MMP25-AS1* successfully counteracted IFN-γ–mediated upregulation of *MMP25* mRNA expression. Furthermore, overexpression of *MMP25-AS1* with both complementary regions intact, 1 of the 2 complementary regions intact, or both complementary regions scrambled verified the ability of *MMP25-AS1* to successfully block IFN-γ–mediated induction of MMP25 protein expression in hSIE cells. The data suggested that both complementary regions, irrespective of size, retained similar capacity to downregulate MMP25 protein expression. Although the presence of 2 complementary regions appears to be redundant, it is possible that both regions may be required for more efficient transcriptional control in nonepithelial cells like neutrophils and other immune cells that showed stronger MMP25 protein expression in the colon (white arrow in Figure 3A). In addition, future studies to delineate the exact mechanisms including the involvement of HuR and HuR/KH-type splicing regulatory protein complexes are needed. RNA pull-down experiments using the full *MMP25-AS1* sequence that contained both complementary regions verified direct physical interactions between *MMP25-ASI* and *MMP25*. Although a previously reported study (56) identified short discontinuous stretches (26 bp) of complementarity between a natural antisense lncRNA and its associated protein-coding gene on the opposite strand, findings from this study, to the best of our knowledge, are the first to report the presence of large continuous stretches (>100 bp) of complementarity between a natural antisense lncRNA and mRNA that can mediate direct suppression of mRNA transcription and protein translation. Taken together, the data suggest that *MMP25-AS1* is a negative regulator of *MMP25* and that low-dose THC administration can epigenetically suppress *MMP25* mRNA expression through enhancement of its natural antisense *MMP25-AS1* expression in intestinal epithelial cells.

Since *MMP25* is known to enhance neutrophil activity (31), we further hypothesized that its elevated expression, partly aided by reduced *TIMP2* and *MMP25-AS1* and enhanced *CCL5* and *CXCL5* expression, will result in increased neutrophil infiltration of the colonic lamina propria. In tune with this postulation, we detected significantly increased infiltration of the colonic lamina propria by MPO/CD11b^++^ activated neutrophils (Figure 6C), suggesting successful transendothelial migration. Interestingly, CD47 protein expression was also significantly elevated in the CE of VEH/SIV RMs (Figure 6D), which indirectly suggested the presence of optimal conditions for neutrophil transepithelial migration. During this process, neutrophils interact with CD47 on the epithelial cells using signal regulatory protein 1-α, causing cytoskeletal changes leading to structural alterations in epithelial cells (57). The migration damages the tight junction proteins, causing widening of the paracellular space, which contributes to barrier disruption and entry of luminal contents (microbial translocation). Interestingly, our data also showed that MMP25 protein expression remained elevated despite viral suppression in the periphery (plasma) and intestine by long-term cART. High MMP25 expression was associated with significantly reduced *MMP25-AS1* expression in JE compared with their respective preinfection time points. Nevertheless, chronic low-dose THC administration in conjunction with cART maintained *MMP25-AS1* expression while preventing *MMP25* upregulation in JE. Although we did not detect changes in MPO/CD11b^++^ neutrophils in the jejunal lamina propria, it is possible that higher frequencies of this neutrophil population may be present in other regions of the small intestine and, more relevantly, the colon in the cART setting, as we have previously shown the colon to be more significantly impacted than the jejunum in HIV/SIV infection (36). However, the colon was not available from the cART group, representing a study limitation. Overall, low-dose THC administration reduced the number of CD11b/MPO^++^ neutrophils and prevented MMP25 and CD47 protein upregulation in the colon. Similar effects on *MMP25* and *MMP25-AS1* expression were also detected in the jejunum when THC was administered in conjunction with cART, thus confirming the intestinal epithelial protective properties of phytocannabinoids in untreated and cART-treated HIV/SIV infection.

**Figure 8:**
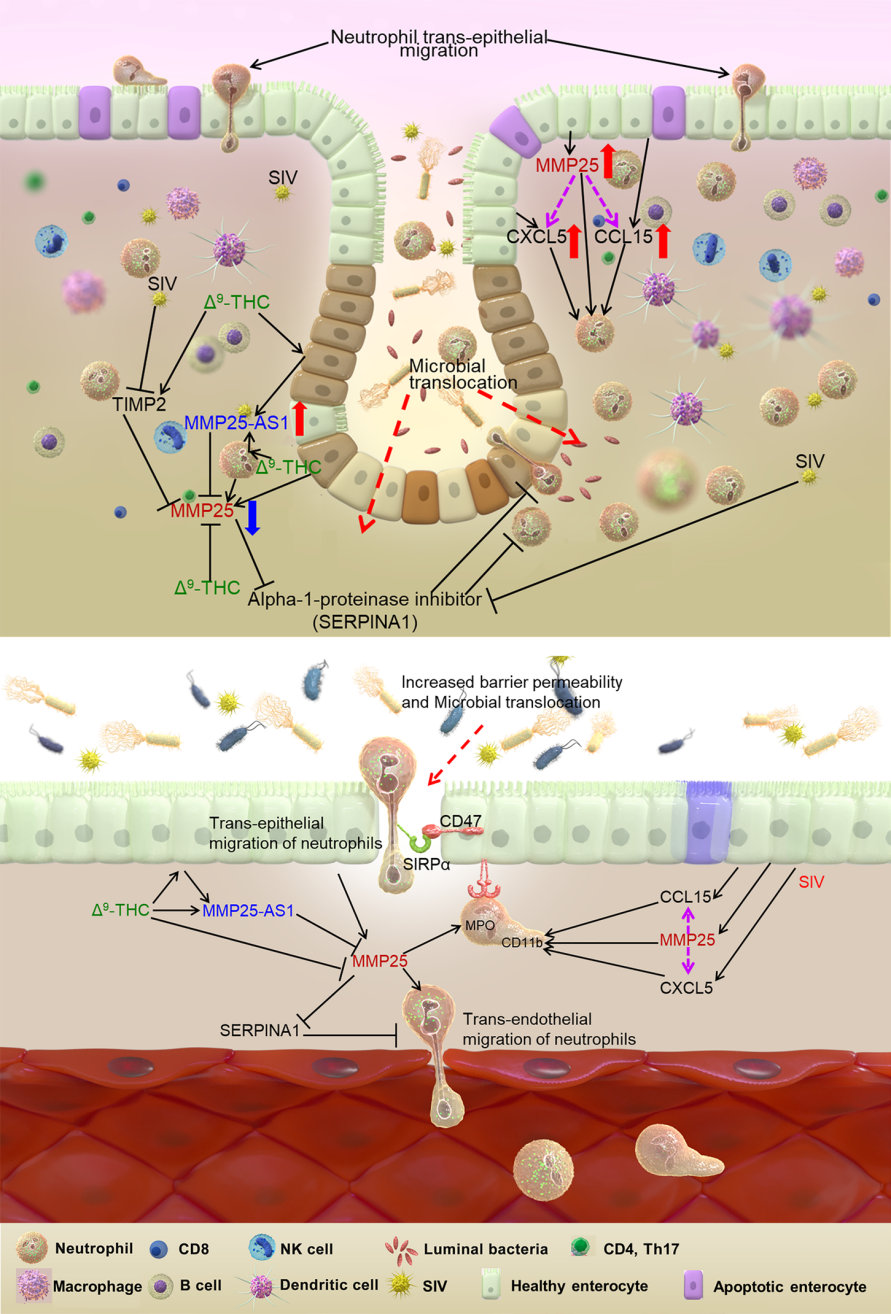
Impact of THC enhancement of *MMP25-AS1/MMP25* interaction on neutrophil transendothelial/transepithelial migration in HIV/SIV infection. At the transcriptional level, *MMP25-AS1* directly interacts with *MMP25*, a matrix metalloproteinase known to enhance the activation of neutrophils and promote inflammation and barrier disruption, allowing microbial translocation. Increased TIMP2 expression results in a further decrease of MMP25 expression at the protein level. The reduction of MMP25 expression also results in a concomitant decrease in CXCL5 and CCL15 expression, 2 potent neutrophil chemoattractants activated by MMP25-mediated cleavage, thus highlighting THC’s potential to block a feed-forward mechanism for neutrophil recruitment. Importantly, THC administration to SIV-infected RMs on long-term cART successfully reduced MMP25 expression in JE of chronically SIV-infected RMs, while concurrently preserving the expression of its natural antisense lncRNA regulator MMP25-AS1. From a therapeutic standpoint, long-term low-dose THC administered as an adjunct to cART represents a potentially safe and effective intervention to dampen chronic immune activation and inflammation, a major driver of HIV-associated comorbidities in virally suppressed PLWH.

In summary, we report findings on changes in the CE lncRNA landscape in response to HIV/SIV infection. Several lncRNAs previously reported to be upregulated in response to inflammatory signaling were DE in CE of VEH/SIV but not THC/SIV RMs. Although we and others have demonstrated the antiinflammatory effects of THC and other cannabinoids in the intestine, the present study goes one step further to demonstrate an epigenetic mechanism involving lncRNAs associated with the intestinal epithelial protective properties of THC. As presented in Figure 8, we describe a molecular mechanism wherein the expression of the natural antisense *MMP25-AS1* upregulated by THC in CE can directly interact with and decrease the expression of *MMP25*. Reduction of *MMP25* expression also resulted in a concomitant decrease in the expression of *CXCL5* and *CCL15*, 2 potent neutrophil chemoattractants activated by MMP25-mediated cleavage (58). These findings not only highlight the role of the intestinal epithelium in neutrophil infiltration through production of MMPs and chemokines but also underscore THC’s potential to block a feed-forward mechanism for neutrophil recruitment. Most importantly, increased *MMP25-AS1* expression was associated with reduced neutrophil infiltration of the colonic lamina propria. From a therapeutic standpoint, long-term, low-dose THC administered as an adjunct to cART successfully reduced MMP25 expression in JE of chronically SIV-infected RMs, while concurrently preserving the expression of *MMP25-AS1*. These encouraging findings will provide the impetus for the development of more effective synthetic THC-like molecules or newer cannabinoid receptor agonists to dampen chronic intestinal inflammation, a major driver of epithelial barrier dysfunction and HIV-associated comorbidities in virally suppressed PLWH. Finally, these translational findings are not restricted to HIV/SIV infection but apply broadly to other chronic inflammatory diseases of the intestine and even lung, where neutrophil transendothelial and transepithelial migration drive pulmonary edema and pathology in conditions like COPD (59).

## Methods

### Experimental design and sample collection.

Twenty-four weight-matched Indian RMs (study performed at the Tulane National Primate Research Center, Covington, Louisiana, USA) were randomly distributed into 3 groups. Group 1 (*n* = 8) animals received twice-daily injections of VEH (1:1:18 of emulphor/alcohol/saline) and were infected intravenously 100 TCID_50_ of SIVmac251 (Table 1). Group 2 (*n* = 9) animals were administered twice-daily injections of THC beginning 4 weeks prior to SIV infection at 0.18 mg/kg as described in our previous studies (15) (Table 1). THC dose was subsequently increased for each animal to 0.32 mg/kg, over a period of approximately 2 weeks, when responding was no longer affected by 0.18 mg/kg on a daily basis (i.e., tolerance developed), and maintained for the duration of the study. Group 3 (*n* = 7) served as uninfected controls (Table 1). The global shortage of Indian RMs, resulting from the unforeseen demand caused by the COVID-19 pandemic (60), made it extremely challenging to find sufficient numbers of SIV-infected male RMs for inclusion in group 1 to achieve statistical significance. Therefore, we included 1 female RM in group 1 (JR17), which had 7 males and 1 female, while groups 2 and 3 comprised only male RMs.

Intact jejunum resection segments (~5 cm) were collected from a separate cohort (Supplemental Table 9) of 16 cART-treated SIV-infected RMs (VEH/SIV/cART; *n* = 8) (THC/SIV/cART; *n* = 8) to verify *MMP25-AS1* and *MMP25* gene expression data initially identified in the colon of cART-naive SIV-infected RMs. cART (PMPA, 20 mg/kg; FTC or emtricitabine, 30 mg/kg; and dolutegravir, 2.5 mg/kg) was initiated at 2 weeks after SIV infection daily by subcutaneous route. We want to mention that 4 macaques each in the VEH/SIV/cART and THC/SIV/cART groups received 2 injections of anti–α_4_β_7_ integrin (LN60, LC48, LM85, LJ21) or control IgG (LM56, LH75, LH92, LI81) (50 mg/kg of anti–α_4_β_7_ or control IgG) beginning 4 MPI at 3-week intervals as part of another study before the jejunum resections were collected at 5 MPI. Our data showed that both anti–α_4_β_7_ and control IgG did not influence the *MMP25-AS1* mRNA (C_T_ values) and MMP25 protein expression (fluorescence intensity) in the jejunum. All animal ages and study schema are shown in Supplemental Table 10 and Supplemental Figure 4, respectively.

At necropsy, colon tissue was collected from all RMs in complete RPMI medium for epithelial cell isolation. Tissues were also collected in RNAlater (Thermo Fisher Scientific) for total RNA extraction. In cART-treated RMs, jejunum resections were collected before and at 5 MPI. For histopathological and immunohistochemical evaluation, colon and jejunum tissues were fixed in Z-fix (Anatech), embedded in paraffin, and sectioned at 7 μM.

### Isolation of intestinal epithelial cells.

For Arraystar microarray and RT-qPCR quantification of lncRNAs and protein-coding genes, epithelial cells from both colon and jejunum were isolated as described previously (61).

### Quantitation of plasma and intestinal viral loads.

Plasma and intestinal viral loads were quantified using RT-qPCR as mentioned in Supplemental Methods.

### Microarray profiling.

Sample preparation and microarray analysis were performed by Arraystar as described in Supplemental Methods.

### GO enrichment analysis.

GO enrichment analysis for DE genes is described in Supplemental Methods.

### Immunofluorescence microscopy.

Immunofluorescence studies for the detection of MMP25 (1:50) (Abcam; ab56309), TIMP2 (1:100) (Novus Biologicals; NBP2-01573), CD47 (1:200) (Mybiosource.com; MBS8106360), MPO (1:6,000) (Abcam; A0398), and CD11b (1:100) (Abcam; ab34216) were performed as described previously (16). MPO and CD11b were stained on the same slide to detect colocalization.

### Quantitative image analysis.

Quantitative analysis of confocal microscopy images was done using HALO (indica labs) as described in Supplemental Methods.

### Plasmid vector construction and overexpression assays.

*MMP25-AS1* plasmid vector constructions for *MMP25-AS1* overexpression assays are described in Supplemental Methods.

To determine the regulatory effect of *MMP25-AS1* on MMP25 protein expression and to determine the contribution of the large and small complementary regions separately on MMP25 protein expression, hSIE cells were cultured in 8-well chambers (Thermo Fisher Scientific) and transfected with all 4 vectors. At 96 hours posttransfection, cells were treated with 50 units of recombinant human IFN-γ and incubated for an additional 24, 48, and 72 hours to determine if *MMP25-AS1* had the ability to counter IFN-γ–mediated induction of MMP25 protein expression. At the respective time points, cells in each chamber were fixed using 2% paraformaldehyde and immunostained to quantify MMP25 protein expression. MMP25 confocal image capture and HALO quantification were performed as described in Supplemental Methods. Data were graphed using GraphPad Prism software.

### RT-qPCR quantification.

To determine the mRNA levels of *MMP25* and *MMP25-AS1* in the jejunum epithelial cells of the cART-treated cohort, RT-qPCR was performed using *MMP25* primers described in the previous section and the following *MMP25-AS1* primers: forward GACAATCGCAGGCCCAGTAA and reverse CTGGCGTGTGAATCCTCTGG. Power SYBR Green RNA to Ct 1-step kit was used (Thermo Fisher Scientific). C_T_ values were obtained and FC expressions were calculated using ΔΔC_T_ method.

### Affinity pull-down of biotinylated RNA.

An affinity pull-down of biotinylated RNA protocol was adapted from a previously published method (62) and as described in Supplemental Methods.

### Data availability.

LncRNA and mRNA profiling data have been submitted to Gene Expression Omnibus (accession number GSE223482; https://www.ncbi.nlm.nih.gov/geo/query/acc.cgi?acc=GSE223482).

### Statistics.

For Arraystar microarray analysis of lncRNA and mRNA, Agilent Technologies Feature Extraction software (version 11.0.1.1) was used to analyze the acquired array images. Quantile normalization and subsequent data processing were performed using GeneSpring GX v12.1 software package (Agilent Technologies). After quantile normalization of the raw data, DE lncRNAs and mRNAs with statistical significance were identified through volcano plot filtering between 2 compared groups. The threshold was FC > 2.0 and *P* < 0.05. FDRs were adjusted from all the *P* values by Benjamini-Hochberg method for multiple-testing correction. Finally, hierarchical clustering was performed using R to show the distinguishable lncRNAs and mRNAs’ expression pattern among samples. For GO enrichment analysis of the DE genes using Gene Ontology resource powered by Panther classification system, the raw *P* values were calculated using Fisher’s exact test. The *q* value (adjusted *P* value, reflecting the FDR) is calculated by the Benjamini-Hochberg procedure. By default, a critical value of 0.05 is used to filter results. Hence, all results shown are valid for an overall FDR. One-way ANOVA (GraphPad, Prism v9) was used for immunofluorescence image quantification analysis of MMP25 (in CE and JE/hSIE cells from overexpression assay), TIMP2, MPO, CD11b, and CD47 (in CE). Shapiro-Wilk test (GraphPad Prism) was used to test for data normality. For *MMP25* RT-qPCR quantification in *MMP25-AS1*–overexpressed hCE and hSIE cells, the *MMP25-AS1*-full sample with the highest ΔC_T_ value served as the calibrator/reference and was assigned a value of 1. *MMP25* RT-qPCR data from hCE were analyzed using unpaired 2-tailed *t* test (GraphPad Prism). *MMP25* RT-qPCR ΔC_T_ values from hSIE cells were analyzed using 1-way ANOVA (GraphPad Prism). For *MMP25* and *MMP25-AS1* RT-qPCR quantification in jejunum epithelial cells, the VEH/SIV/cART RM with the highest ΔC_T_ value served as the reference and assigned a value of 1. Gene expression data are shown as a fold difference relative to these 2 reference samples in their respective experiments. ΔC_T_ values were analyzed using 1-way ANOVA (GraphPad Prism).

### Study approval.

All experiments using RMs were approved by the Tulane University Institutional Animal Care and Use Committee (Protocol s3581/3781, Covington, Louisiana, USA). All clinical procedures, including administration of anesthesia and analgesics, were carried out under the direction of a laboratory animal veterinarian (AAALAC 000594).

## Author contributions

MM and LSP carried out the overall planning, direction, and design of the experiments. LSP, MMW, EL, and MM carried out the day-to-day sampling and scheduling (animal experiments) and performed the RT-qPCR validation of lncRNAs and genes of interest, immunofluorescence/image quantification, in vitro overexpression experiments, and data analysis. SK and SJ performed the RNA pull-down experiments and assisted with *MMP25-AS1* overexpression and isoform validation studies. XA assisted with confocal image capture and HALO image analysis. SNB assisted with plasma and tissue viral load assays. BL and CMO assisted with the cell culture, data analysis, and conclusions. LSP and MM wrote the manuscript with input from all authors. MMW, BL, SNB, CMO, and SK provided helpful suggestions and review of the manuscript. All authors read and approved the final version of the manuscript.

## Supplementary Material

Supplemental data

## Figures and Tables

**Table 1:**
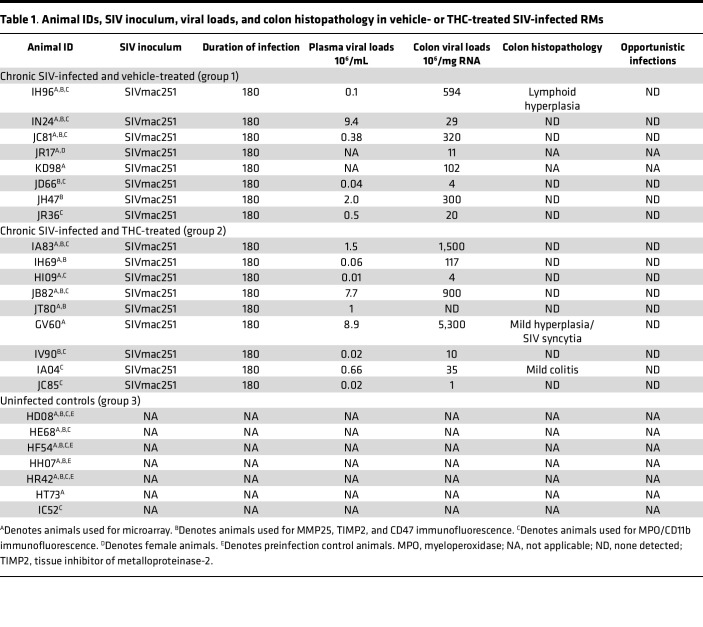
Animal IDs, SIV inoculum, viral loads, and colon histopathology in vehicle- or THC-treated SIV-infected RMs

## Version 1 04/10/2023

Electronic publication
