# Supplementary material for: Cannabinoid enhancement of lncRNA MMP25-AS1/MMP25 interaction reduces neutrophil infiltration and intestinal epithelial injury in HIV/SIV infection
Source: JCI Insight. 2023 Apr 10;8(7):e167903. doi: 10.1172/jci.insight.167903 (PMC10132162; doi:10.1172/jci.insight.167903)

Supplemental Materials for

Cannabinoid enhancement of lncRNA-*MMP25-AS1/MMP25* interactions reduces neutrophil infiltration and intestinal epithelial injury in HIV/SIV infection

Lakmini S Premadasa[1], Eunhee Lee[1] , Marina McDew-White[1], Sahana Jayakumar [1], Binhua Ling[1], Chioma M Okeoma[2], Siddappa N Byrareddy[3], Smita Kulkarni[1], Mahesh Mohan[1*]

*Corresponding author. Email: mmohan@txbiomed.org

This PDF file includes:

Supplemental methods

Supplemental Figure legends

Supplementary Tables 1 to 10

Supplementary Figures 1 to 4

Supplemental methods

Quantitation of plasma and intestinal viral loads

Total RNA samples from all SIV-infected animals were subjected to a quantitative real-time TaqMan One-step RT-qPCR analysis to quantify SIV viral RNA copies in plasma and whole intestinal tissues. Briefly, primers and probes specific to the SIV LTR sequence were designed and used in the real-time TaqMan PCR assay. Probes were conjugated with a fluorescent reporter dye (FAM) at the 5′ end and a quencher dye at the 3′ end. Fluorescence signal was detected with a Quantstudio 5 Real-Time PCR system (Thermo Fisher). Data were captured and analyzed with QuantStudio Design & Analysis Software (Thermo Fisher). Viral copy number was determined by plotting C_T values obtained from plasma and colon samples against a standard curve ($y=-3.315x + 41.429$) ($r^2=0.999$) generated with *in vitro* transcribed RNA representing known viral copy numbers. Samples with C_T values >37.5 were considered as non-detected.

Microarray profiling of lncRNAs and mRNAs

Sample preparation and microarray analysis was performed by Arraystar Inc. (Rockville MD, USA). Arraystar Human LncRNA Microarray v4.0 is designed for the global expression profiling of human lncRNA and protein-coding mRNA transcripts. The array detects a total of 40,173 lncRNAs in two tiered compilations: Gold Standard lncRNAs for 7,506 well annotated, functionally studied and experimentally supported full length lncRNAs, and reliable lncRNAs for 32,667 high confidence lncRNAs as the comprehensive collection. The array also includes a large collection of 20,730 protein coding mRNAs supported by UniProt (Universal Protein Resource) catalog. Each transcript is represented by a specific exon or splice junction probe, which uniquely identifies individual transcripts accurately. Positive probes for housekeeping genes and negative

probes are printed on the array to serve as hybridization quality controls. More importantly, owing to their generally low to very low expression levels, the microarray platform designed to capture both poly(A) and non-poly(A) lncRNAs is preferred over conventional RNA-seq.

Total RNA from each sample was quantified by the NanoDrop ND-1000 and RNA integrity was assessed by standard denaturing agarose gel electrophoresis. For microarray analysis, Agilent Array platform was employed. Sample labeling and array hybridization were performed according to the Agilent One-Color Microarray-Based Gene Expression Analysis protocol (Agilent Technology) with minor modifications. Each sample was amplified and transcribed into fluorescent cRNA along the entire length of the transcripts without 3' bias utilizing a random priming method (Arraystar Flash RNA Labeling Kit, Arraystar). The labeled cRNAs were purified using RNeasy Mini Kit (Qiagen). The concentration and specific activity of the labeled cRNAs (pmol Cy3/μgcRNA) were measured using NanoDrop ND-1000. About 1 μg of each labeled cRNA was fragmented by adding 5 μl 10x blocking agent and 1 μl 25x fragmentation buffer, then heated to 60 °C for 30 min, finally 25 μl 2 × GE hybridization buffer was added to dilute the labeled cRNA. 50 μl of hybridization solution was dispensed into the gasket slide and assembled to the lncRNA expression microarray slide. The labeled cRNAs were hybridized onto the human lncRNA array v4.0 (8 x 60K, Arraystar). The slides were incubated for 17 h at 65 °C in an agilent hybridization oven. The hybridized arrays were washed, fixed and scanned using the agilent DNA microarray scanner (part number G2505C).

GO enrichment analysis

Gene Ontology (GO) enrichment analysis for DE genes to determine biological processes and functions was carried out using Gene Ontology resource (Gene Ontology Resource) powered by Panther classification system (www.pantherdb.org). Gene lists were entered with gene IDs and

homo sapiens was selected as the organism. Number of genes expressed for selected biological processes were recorded for both THC/SIV vs control and VEH/SIV vs control and compared.

Quantitative image analysis of intestinal sections

Briefly, two slides containing colon or jejunum tissue sections from each animal were stained with antibodies specific for MMP25, TIMP2, MPO, CD11b, and CD47. No differences in staining intensity were detected between slides for each macaque. A total of ten bright-field sections for each rhesus macaque (from groups 1, 2 and 3) scanned using a Zeiss LSM700 confocal microscope (Carl ZEISS Microscopy, LLC) at 20X objective. Digital images were imported into HALO software (Indica Labs) for image quantitation analysis. Since colonic epithelial cells were used for lncRNA and mRNA profiling, we first demarcated the epithelial regions in both colon and jejunum as shown in Fig. 3b, and accordingly used the area quantification module available on HALO v3.2 (Indica Labs) to quantify MMP25, CD47 (green signal/Alexa-488) and, TIMP2 (red signal/Alexa-568) fluorescence from both tissues. For MPO and CD11b quantitation, the highplex FL module on HALO was used. In this new computational method, the artificial intelligence driven software identifies all cells that express MMP25, CD11b and CD47 in green, TIMP2 and MPO in red and nuclei in blue (DAPI) and also categorizes the cells based on predefined fluorescence intensity levels. Specifically, the HALO software normalizes the threshold across all images and enables quantitation of the number of cells, and relative intensity of fluorescence per cell, of single channel fluorescence (green or red) corresponding to the expression of MMP25, TIMP2, MPO, CD11b, and CD47 that was intensely expressed in the intestinal epithelial (MMP25, TIMP2, CD47) and lamina propria cells (MPO and CD11b). The output values (total area and average positive intensity) were used to calculate the total MMP25,

TIMP2, MPO, CD11b, and CD47 fluorescent intensity/tissue area. The data were graphed using Prism v9 software (GraphPad software).

Plasmid vector construction and overexpression assays

To determine the impact of *MMP25-AS1* lncRNA on *MMP25* mRNA and protein expression, we overexpressed *MMP25-AS1* in human colonic epithelial (hCE) (Sciencell research laboratories, Carlsbad, CA, USA; Catalog #2950) and human small intestinal epithelial (hSIE) cells (ATCC research laboratories, Manassas, VA, USA; Cat #CRL-3266). For *MMP25-AS1* overexpression studies, four different pcDNA3.1 vectors containing the full-length *MMP25-AS1* sequence with both complementary regions intact (MMP25-AS1-full), *or* only the large complementary region intact (*MMP25-AS1*-LI), or small complementary region intact (*MMP25-AS1*-SI), or with both complementary regions scrambled (*MMP25-AS1*-BS) were synthesized by GENEWIZ/Azenta (South Plainfield, NJ, USA). hSIE cells were transfected with all four *MMP25-AS1* vectors using lipojet transfection reagent (Signagen, MD) in a 24-well plate and cultured for 120h (transfection efficiency>95%). In addition to determining the interactions between *MMP25-AS1* lncRNA and *MMP25* mRNA, *MMP25-AS1* transfected cells were also treated with 2µg/mL LPS (Sigma Aldrich) or 50 units of human recombinant IFNγ (Biotechne) to identify a proinflammatory stimuli responsible for its induction *in vivo*. At 96h post transfection, cells were treated with either LPS or IFNγ and 24h later cells were lysed and total RNA was extracted using the RNeasy Mini Kit (Qiagen). Quantitative real-time One-step RT-qPCR to quantify *MMP25* mRNA was performed using the Power SYBR Green RNA to C_T 1-step kit (Applied biosystems catalog #01236971) using the following primers; Forward – 5' ACAGATCAGCATGAGGACAG 3' Reverse – 5' ACTGACAGAGGCCCAAATC 3'. Fluorescence signal was detected with a

Quantstudio 5 Real-Time PCR system (Thermo Fisher). C_T values were obtained and fold change was calculated using ΔΔCT method.

Affinity pulldown of biotinylated RNA and RT-qPCR analyses

An affinity pulldown of biotinylated RNA protocol was adapted from a previously published method (70). Full-length *MMP25-AS1* (2349bp) transcripts were in vitro transcribed and biotinylated using MEGAscriptTM T7 transcription kit (Thermo fisher Scientific). A second lncRNA, (SLNCR1 alias LINC00673) of similar nucleotide length (2257bp) was in vitro transcribed, biotinylated and used as a negative control. hSIE cells were lysed in the presence of protease and RNAse inhibitors. Biotinylated RNAs were mixed with the lysate and incubated at room temperature for 30 minutes. LncRNA-mRNA complexes were isolated and purified using streptavidin coated magnetic beads. Fold enrichment of *MMP25-AS1, LINC00673* and *MMP25* transcripts along with the housekeeping genes was analyzed using RT-qPCR using previously described primers for *MMP25* and *MMP25-AS1* and the following primers for *LINC00673*; forward – 5’ GAGAACGTGGTGGAATCAGA 3’, reverse – 5’ TCCCATCCTCTTTCTTGTCC 3’.

Supplemental Figure legends

Supplemental Figure 1. Complementary interaction between human *MMP25-AS1* lncRNA and *MMP25* mRNA. Screenshot of CLUSTALW analysis of *MMP25* mRNA and *MMP25-AS1* lncRNA showing small (133bp) and large (374bp) complementary regions (A).

Supplemental Figure 2. Interaction between macaque *MMP25 mRNA – human MMP25-AS1 lncRNA* and *human MMP25-AS1 – macaque MMP25-AS1*. Sequencing results of the PCR product (amplified using primer pair 1 in Figure 4F) from macaque colonic epithelial cells show the presence of *MMP25-AS1* isoform ENST00000572574.5 (A). Screenshot of CLUSTALW complementary analysis of predicted macaque *MMP25* mRNA and human *MMP25-AS1* showing small and large complementary regions (B). Screenshot of CLUSTALW homology analysis of human MMP25-AS1 isoform ENST00000572574.5 and PCR amplified MMP25-AS1 sequence from CE of macaque (C) showing perfect (99%) homology.

Supplemental Figure 3. *MMP25-AS1* overexpression reduces MMP25 protein expression in hSIE cells. MMP25 protein expression in hSIE cells overexpressing *MMP25-AS1*-BS (n=4) or *MMP25-AS1*-full (n=4) or *MMP25-AS1*-SI (n=4) or *MMP25-AS1*-LI (n=4) in response to IFNγ treatment at 24h, 48 h and 72h post-IFNγ treatment (A). Immunofluorescence images were captured using a Zeiss confocal microscope at 20X magnification. MMP25 signal intensity was Quantified using Halo software (Highplex FL method). Differences in MMP25 signal intensity between treatment groups were analyzed using one-way ANOVA employing the Prism v9 software (GraphPad prism) (B, C). A p-value of <0.05 was considered significant. Data represent mean±SEM.

Supplemental Figure 4. Schematic representation of the study design

Two separate RM cohorts (cART naïve and cART experienced) were administered THC (0.18mg/Kg) for 4 weeks before the SIV infection and continued (at 0.32mg/Kg) until necropsy. Colon tissues were collected at necropsy from cART naïve cohort (VEH/SIV, n=5), (THC/SIV, n=6), (uninfected controls, n=6). cART experienced cohort (VEH/SIV/cART, n=8), (THC/SIV/cART, n=8) was given Emtricitabine/Tenofovir/Dolutegravir at 2 weeks post infection and continued until 5MPI. Jejunum surgical resections were collected at -1-month (pre-SIV-infection) and 5MPI from the cART cohort.

Supplemental Tables

Supplemental table 1. Important lncRNAs upregulated in VEH/SIV compared to uninfected control RMs (*PCG – Protein coding gene)

lncRNA Gene Symbol	P Value	Fold Change	Relationship	Associated PCG Name
CALML4	0.00004	7.4	exon sense- overlapping	CALML4
FOXP4-AS1	0.00031	5.5	bidirectional	FOXP4
BISPR	0.00046	6.7	exon sense- overlapping	MVB12A
MLLT4-AS1	0.00051	4.2	bidirectional	MLLT4
SNHG18	0.002	6.2	bidirectional	SEMA5A
GATA6-AS1	0.01304	5.9	bidirectional	GATA6
HOTAIRM1	0.0191	3	bidirectional	HOXA1
IDH1-AS1	0.00028	2.3	intronic antisense	IDH1
HNF4A-AS1	0.01254	2.7	intronic antisense	HNF4A
PTPRG-AS1	0.01361	4.7	natural antisense	PTPRG
SPRY4-IT1	0.03809	2.6	intron sense- overlapping	SPRY4

Supplemental table 2. Important LncRNAs downregulated in VEH/SIV compared to uninfected control RMs

lncRNA Gene Symbol	P Value	Fold Change	Relationship	Associated PCG Name
CTB-147C22.9	0.00021	2.5	intronic antisense	KLK9
CTB-147C22.9	0.00021	2.5	intronic antisense	KLK8
NEURL1-AS1	0.00081	163.9	intronic antisense	NEURL1
HOXA-AS2	0.00114	119.1	natural antisense	HOXA4
AC067945.4	0.0032	3	natural antisense	STAT4
RP1-71H24.1	0.00388	7.7	natural antisense	OAS1
AC078883.4	0.00411	6	natural antisense	ITGA6
SOCS2-AS1	0.00496	2.7	intronic antisense	SOCS2
RAD51-AS1	0.00583	3.5	natural antisense	RAD51
ABALON	0.00626	11.8	natural antisense	BCL2L1
XIAP-AS1	0.00802	7.4	intronic antisense	XIAP
LOC101929512	0.00868	8.9	intronic antisense	TANK
OGFR-AS1	0.0088	4.6	intronic antisense	OGFR
SDCBP2-AS1	0.00959	8.1	intronic antisense	FKBP1A
LIPE-AS1	0.01044	3	intronic antisense	CEACAM1
PARD3-AS1	0.01736	3.1	bidirectional	PARD3
RP11-104J23.1	0.01957	3.8	natural antisense	CCL15
CCL15-CCL14	0.02006	2.7	exon sense- overlapping	CCL15
AC004837.5	0.03631	6	intronic antisense	RALA
HOXC-AS2	0.04744	2.1	intronic antisense	HOXC6

Supplemental table 3. Important LncRNAs Upregulated in THC/SIV compared to uninfected control RMs

lncRNA Gene Symbol	P Value	Fold Change	Relationship	Associated PCG Name
BISPR	3.24138E-06	15.6	exon sense-overlapping	MVB12A
SLMO2-ATP5E	4.23611E-06	3.4	exon sense-overlapping	ATP5E
THAP9-AS1	0.00003	4.7	exon sense-overlapping	SEC31A
MLLT4-AS1	0.00034	3.4	bidirectional	MLLT4
LINC01436	0.00072	3.4	bidirectional	RUNX1
NEAT1	0.00348	2.6	intergenic	
PTPRD-AS1	0.00432	3.1	intronic antisense	PTPRD
LOC100505716	0.01478	3.1	intronic antisense	BRE
HOTAIRM1	0.01515	2.4	bidirectional	HOXA1
XLOC_011197	0.04866	3	intronic antisense	TJP1
HOXA-AS3	1.03555E-07	2.4	intronic antisense	HOXA5
LOC100128593	3.95171E-06	7	natural antisense	LCN6
HOXA-AS3	1.03555E-07	2.4	natural antisense	HOXA7
GK-AS1	0.00035	6.1	natural antisense	GK
RORA-AS1	0.00111	2.4	intronic antisense	RORA
IFNG-AS1	0.00593	4.7	intergenic	IFNG
RP11-322D14.2	0.01119	2.7	intronic antisense	NLRC5
ZFAS1	0.01353	2.1	natural antisense	ZNFX1

Supplemental table 4. Important LncRNAs Downregulated in THC/SIV compared to uninfected control RMs

lncRNA Gene Symbol	P Value	Fold Change	Relationship	Associated PCG Name
INAFM1	9.97003E-07	80.4	exon sense-overlapping	PRR24
RP11-15E18.1	0.00001	6.8	bidirectional	APPBP2
G059911	0.00008	3.2	bidirectional	MRFAP1
LGALS8-AS1	0.00025	5.7	intronic antisense	LGALS8
AK022061	0.00032	7	bidirectional	DENND4C
AC078883.4	0.00064	6.9	natural antisense	ITGA6
RP1-71H24.1	0.00072	9.5	natural antisense	OAS1
RP11-149I9.2	0.00074	5.5	intronic antisense	AATK
RP11-326I11.3	0.00121	5.2	bidirectional	IRF2
G086201	0.00143	4	bidirectional	TXN
RP11-104J23.1	0.00148	5.6	natural antisense	CCL15
CTB-147C22.9	0.00177	2.1	intronic antisense	KLK8
CTB-147C22.9	0.00177	2.1	intronic antisense	KLK9
LOC256880	0.00189	6.4	bidirectional	H2AFZ
uc.350	0.00393	4.5	intronic antisense	DACH1
OGFR-AS1	0.00518	4.4	intronic antisense	OGFR
RAD51-AS1	0.00637	3.5	natural antisense	RAD51

G007771	0.00846	2.6	intronic antisense	AKT3
AC007078.4	0.01065	3.2	intronic antisense	DTX2

Supplemental table 5. Important LncRNAs Upregulated in VEH/SIV compared to THC/SIV RMs

lncRNA Gene Symbol	P Value	Fold Change	Relationship	Associated PCG Name
FOXP4-AS1	0.00225	2	bidirectional	FOXP4
RP11-165A20.3	0.00388	2.8	bidirectional	PRTFDC1
TRIM36-IT1	0.01025	2.2	intron sense-overlapping	TRIM36
HNF4A-AS1	0.01211	2.1	intronic antisense	HNF4A
GATA6-AS1	0.02527	4.4	bidirectional	GATA6
RP11-761I4.4	0.02777	2.8	natural antisense	IL16
G040952	0.02948	2.3	natural antisense	BCL3

Supplemental table 6. Important LncRNAs downregulated in VEH/SIV compared to THC/SIV RMs

lncRNA Gene Symbol	P Value	Fold Change	Relationship	Associated PCG Name
RAMP2-AS1	0.00092	49.3	natural antisense	RAMP2
PGM5-AS1	0.00461	16.9	intronic antisense	PGM5
HOXA-AS2	0.00489	25.7	natural antisense	HOXA4
ZEB2-AS1	0.00594	17.2	natural antisense	ZEB2
NEURL1-AS1	0.0144	15.4	intronic antisense	NEURL1
G016487	0.01566	11.2	natural antisense	RAD52
G027485	0.01758	2.2	intronic antisense	TNFAIP8L3
CCL4L1	0.01776	2.9	exon sense-overlapping	CCL4L2
CTD-2373H9.5	0.02448	8.5	intronic antisense	TIMP2
BISPR	0.03462	2.3	exon sense-overlapping	MVB12A
IFNG-AS1	0.04383	2.8	intergenic	IFNG

Supplemental table 7. List of down-regulated natural antisense lncRNAs associated with the up-regulation of their corresponding protein coding genes in CE of THC/SIV RMs

Gene symbol (lncRNA)	Regulation (lncRNA)	P-Value (lncRNA)	Fold change (lncRNA)	Associated PCG name	Regulation (PCG)	P-Value (PCG)	Fold change (PCG)
G040065	down	0.001028627	14.5286467	C19orf12	up	0.039017377	3.1820241
AC078883.4	down	0.000640609	6.8628166	ITGA6	up	0.040958467	2.0855422
G063378	down	0.00106922	6.1843948	FBXW7	up	0.01470242	2.5109857
G030931	down	4.10543E-05	6.0319823	N4BP1	up	0.000168404	2.8656698
RP11-1079K10.4	down	0.020417724	5.7449591	PHB	up	0.011407167	2.7847042
LGALS8-AS1	down	0.00024532	5.7210572	LGALS8	up	0.000414467	3.7657026
G071932	down	0.007594866	5.6559045	SYNCRIP	up	0.044591555	2.0126263
RP11-104J23.1	down	0.001475241	5.6301291	CCL15	up	0.001471283	3.6768664
G015336	down	0.000172495	4.8931636	ARHGAP42	up	0.007921877	2.1597814
JX088243	down	0.043356203	4.7532958	IGF1	up	0.003895315	6.0820336
RP5-864K19.4	down	0.026922806	4.1642005	RHBDL2	up	0.009001956	2.3691545

G011378	down	0.001099522	4.0048182	VTI1A	up	0.000182216	2.474389
RP11-875O11.1	down	0.00064941	3.5388481	RHOBTB2	up	0.000347745	3.5281708
G065567	down	0.031858741	3.4045762	HMGCS1	up	0.000283444	5.8636901
MAGI2-AS3	down	0.046776105	3.2441887	MAGI2	up	0.026131522	2.7518337
G050192	down	0.00701774	2.6510636	SPAG4	up	1.39863E-06	10.7493808
LOC100506083	down	0.004897657	2.4989629	DDX19B	up	0.023722967	2.4682777
DARS-AS1	down	0.00367852	2.3441569	DARS	up	0.002669115	2.3435453
G085853	down	0.011508845	2.2291195	XPA	up	0.039322482	3.0539717
JMJD1C-AS1	down	0.014021448	2.2287952	JMJD1C	up	0.041362801	2.1307478
CTD-2145A24.4	down	0.007317776	2.2074322	PRPSAP2	up	0.00620796	2.1996047
RP11-323J4.1	down	0.04843706	2.2026748	RAP1GDS1	up	0.00393756	2.3910292
DHRS4-AS1	down	0.020011819	2.1943235	DHRS4	up	0.000203161	2.6293991
G014822	down	0.004861785	2.1192972	ACER3	up	0.000567962	2.1478084
uc.346	down	0.006340728	2.0864064	RFX4	up	0.000355834	2.5691806
PLBD1-AS1	down	0.009788594	2.0300407	PLBD1	up	0.014939668	2.3094503

Supplemental table 8. List of up-regulated natural antisense lncRNAs associated with the down-regulation of their corresponding protein coding genes in CE of THC/SIV RMs

Gene symbol (lncRNA)	Regulation (lncRNA)	P-Value (lncRNA)	Fold change (lncRNA)	Associated PCG name	Regulation (PCG)	P-Value (PCG)	Fold change (PCG)
RP4-724E13.2	up	0.002355385	3.2068408	COBL	down	0.040058936	2.0279774
ASMTL-AS1	up	4.22E-05	3.1104695	ASMTL	down	0.003762708	2.2462088
G015934	up	0.003293354	2.9897422	OAF	down	0.0036127	3.3025435
RP11-127L20.5	up	0.00031502	2.9495083	ITPRIP	down	9.54E-07	2.7996271
G066388	up	0.020791685	2.4852387	OTP	down	0.033462974	2.0732651
MMP25-AS1	up	0.0420752	2.4060153	MMP25	down	0.00085916	2.2033227
LOC100133077	up	0.007517507	2.3074728	CACNA1B	down	0.01330658	2.4180967
RP1-50J22.4	up	0.001232109	2.0901676	PXT1	down	1.33E-06	7.6330287

Supplemental table 9. Animal IDs, SIV inoculum, viral loads and jejunum histopathology in cART treated SIV-infected RMs.

Animal ID	SIV Inoculum	Duration of Infection	Plasma viral loads 10^6/mL	Jejunum viral loads 10^6/mg RNA	Jejunum Histopathology	Opportunistic Infections
**Chronic SIV-Infected, cART and Vehicle treated**						
LA88	SIVmac251	150	ND	ND	ND	ND
LC39	SIVmac251	150	ND	ND	ND	ND
LD08	SIVmac251	150	ND	ND	ND	ND
LE67	SIVmac251	150	ND	ND	ND	ND
LM56	SIVmac251	150	ND	ND	ND	ND
LN60	SIVmac251	150	ND	ND	ND	ND
LH75	SIVmac251	150	ND	ND	ND	ND
LC48	SIVmac251	150	ND	ND	ND	ND
**Chronic SIV-Infected, cART and Δ^9-THC treated**						
LA55	SIVmac251	150	ND	ND	ND	ND
LB61	SIVmac251	150	ND	ND	ND	ND
LA89	SIVmac251	150	ND	ND	ND	ND
KV50	SIVmac251	150	ND	ND	ND	ND
LM85	SIVmac251	150	ND	ND	ND	ND
LH92	SIVmac251	150	ND	ND	ND	ND
LJ21	SIVmac251	150	ND	ND	ND	ND
LI81	SIVmac251	150	ND	ND	ND	ND

ND- None detected

Note: All 16 animals had jejunum resections (~5 cm piece) collected before (Pre-infection controls) and again at 5 months post SIV infection

Supplemental table 10. Ages of SIV-infected animals administered either THC or VEH and uninfected controls

**Animal ID**	**Age (Years)**
**Chronic SIV-Infected, Vehicle treated**	
IH96	4.98
IN24	5.66
JC81	4.81
JR17	6.04
KD98	5.26
JD66	7.56
JH47	7.38
JR36	6.46
**Chronic SIV-Infected, THC treated**	
IA83	5.84
IH69	5.02
HI09	7.59
JB82	4.78
JT80	6.29
GV60	7.17
IV90	7.69
IA04	6.61
JC85	7.52
**Uninfected control**	
HD08	6.25
HE68	6.23
HF54	6.42
HH07	6.35
HR42	5.5
HT73	4.16
IC52	3.88

**Animal IDs**	**Age (Years)**
**Chronic SIV-Infected, cART, Vehicle treated**	
LA88	4.65
LD08	4.56
LC39	4.61
LE67	4.48
LM56	3.65
LC48	5.71
LH75	4.66
LN60	3.60
**Chronic SIV-Infected, cART, THC treated**	
LA55	4.68
LB61	4.64
LA89	4.64
KV50	5.30
LM85	3.64
LH92	4.70
LJ21	4.50
LI81	4.58

Supplemental Figures

Supplemental Figure 1. Complementary interaction between human *MMP25-AS1* lncRNA and *MMP25* mRNA.

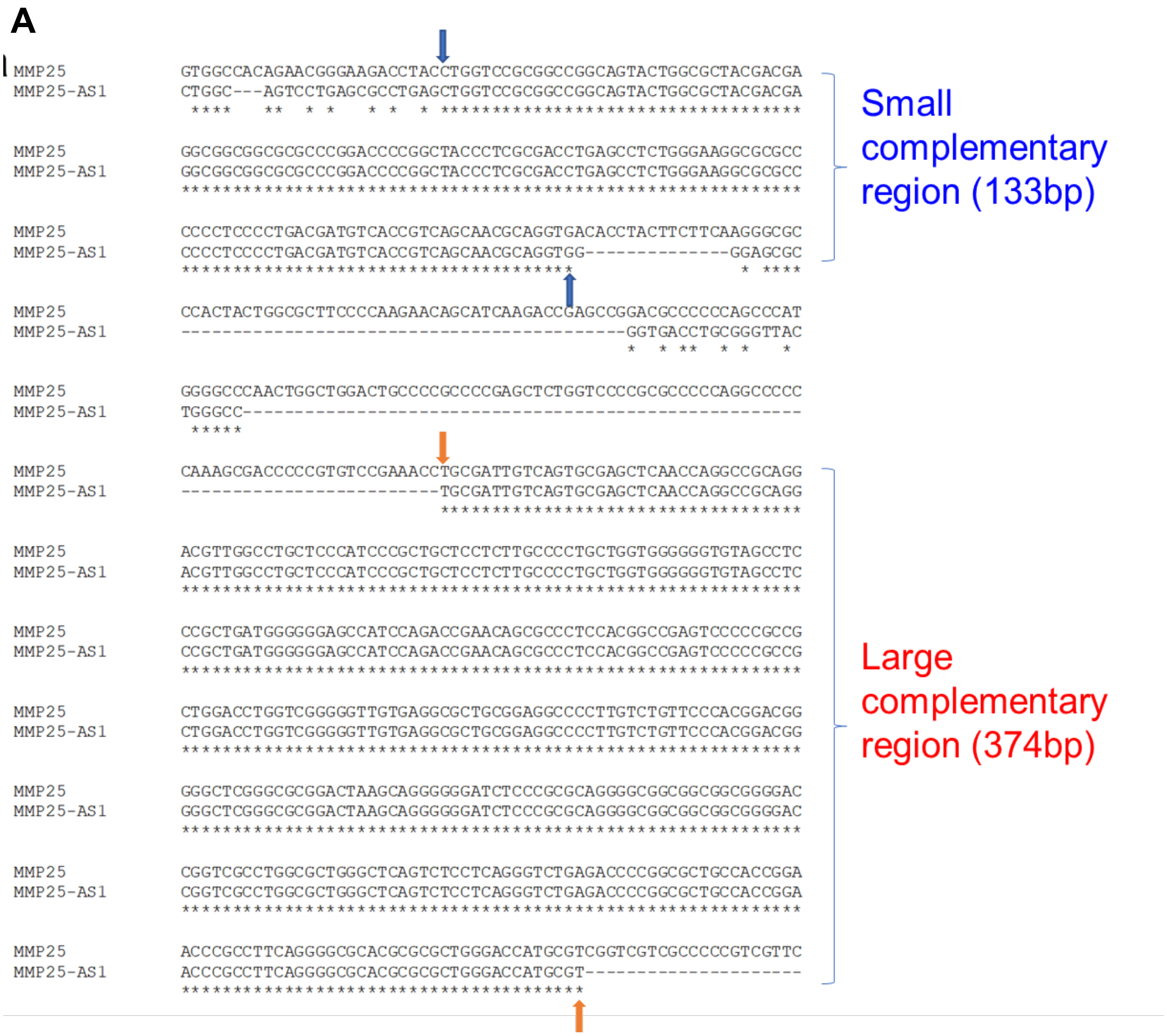

Supplemental Figure 2. Interaction between macaque *MMP25 mRNA* – human *MMP25-AS1 lncRNA* and human *MMP25-AS1* – macaque *MMP25-AS1*.

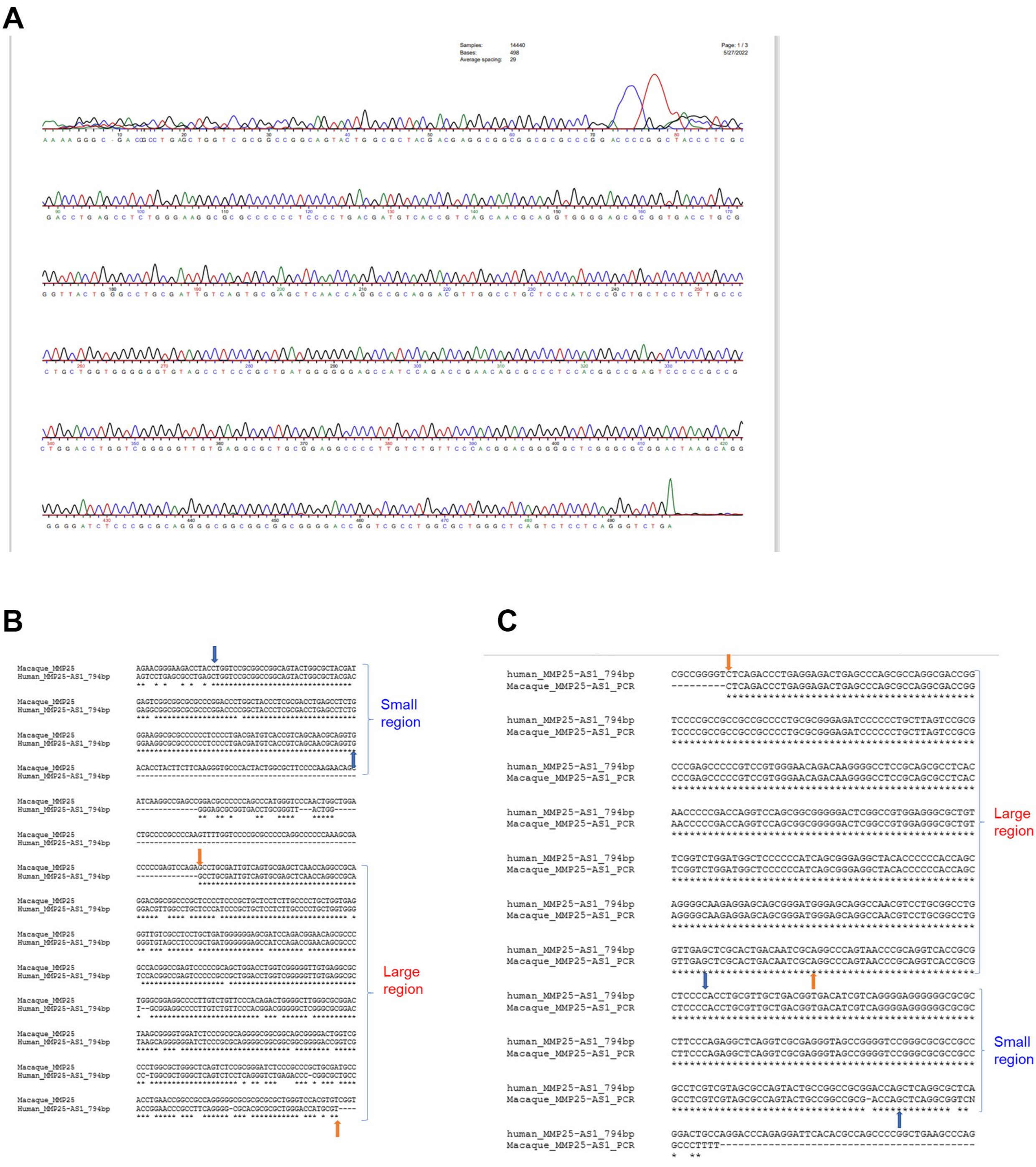

Supplemental Figure 3. ***MMP25-AS1*** **overexpression reduces MMP25 protein expression in hSIE cells.**

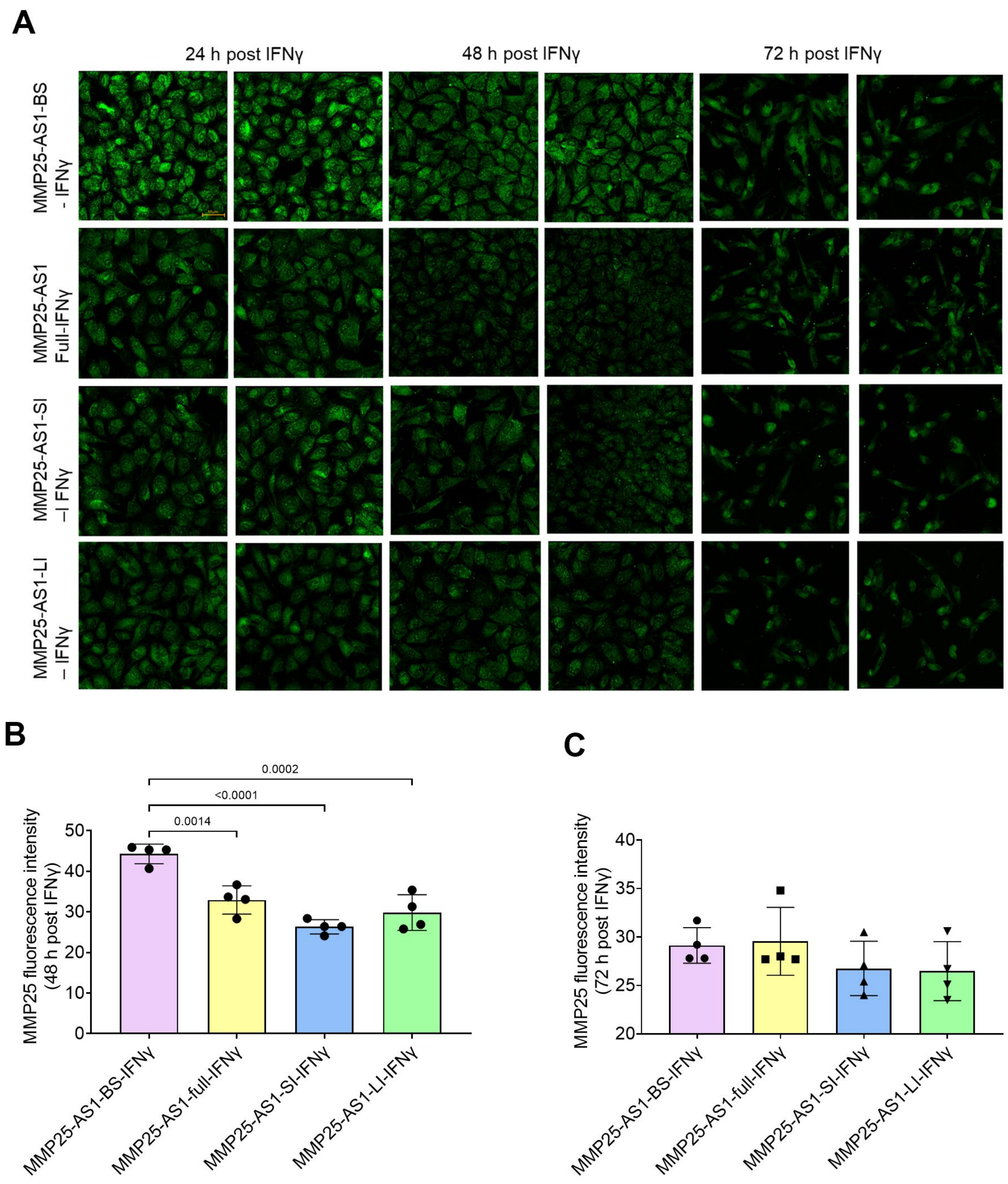

Supplemental Figure 4. Schematic representation of the study design

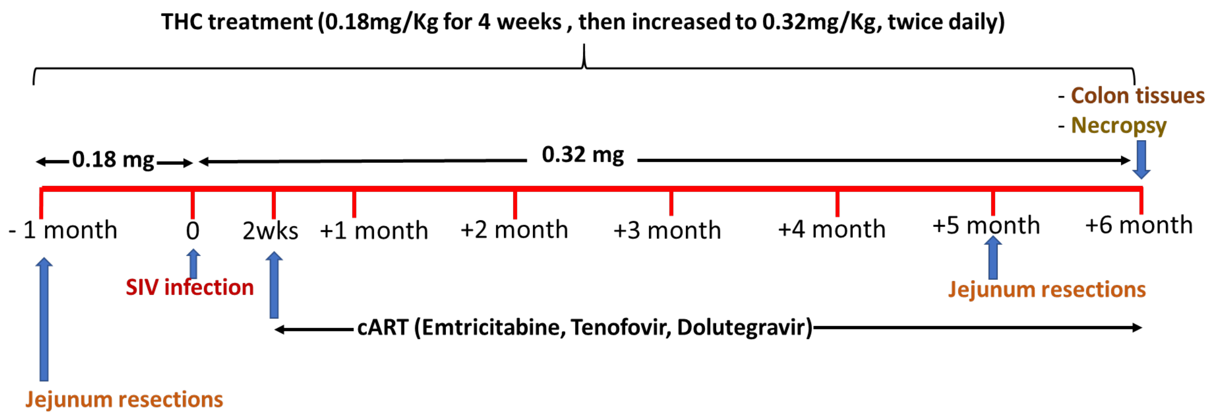

Supplement: Supplemental data [file jciinsight-8-167903-s185.pdf]
